# Dual anti-angiogenic and anti-inflammatory action of tRNA-Cys-5-0007 in ocular vascular disease

**DOI:** 10.1186/s12967-024-05338-w

**Published:** 2024-06-12

**Authors:** Yan Ma, Ying Zhang, Hui-Ying Zhang, Ya Zhao, Xiu-Miao Li, Yi-Fei Jiang, Mu-Di Yao, Qin Jiang, Biao Yan

**Affiliations:** 1 The Fourth School of Clinical Medicine, Nanjing Medical University, Nanjing, 210000 China; 2 The Affiliated Eye Hospital, Nanjing Medical University, Nanjing, 210000 China; 3 Department of Ophthalmology, Shanghai General Hospital, Shanghai Jiao Tong University School of Medicine, Shanghai, 200080 China

**Keywords:** Ocular vascular disease, Exosomal formulation, Transfer RNA-derived fragment, Anti-angiogenic therapy

## Abstract

**Background:**

Intravitreal injections of angiogenesis inhibitors have proved efficacious in the majority of patients with ocular angiogenesis. However, one-fourth of all treated patients fail to derive benefits from intravitreal injections. tRNA-derived small RNA (tsRNA) emerges as a crucial class of non-coding RNA molecules, orchestrating key roles in the progression of human diseases by modulating multiple targets. Through our prior sequencing analyses and bioinformatics predictions, tRNA-Cys-5-0007 has shown as a potential regulator of ocular angiogenesis. This study endeavors to elucidate the precise role of tRNA-Cys-5-0007 in the context of ocular angiogenesis.

**Methods:**

Quantitative reverse transcription PCR (qRT-PCR) assays were employed to detect tRNA-Cys-5-0007expression. EdU assays, sprouting assays, transwell assays, and Matrigel assays were conducted to elucidate the involvement of tRNA-Cys-5-0007 in endothelial angiogenic effects. STZ-induced diabetic model, OIR model, and laser-induced CNV model were utilized to replicate the pivotal features of ocular vascular diseases and evaluate the influence of tRNA-Cys-5-0007 on ocular angiogenesis and inflammatory responses. Bioinformatics analysis, luciferase activity assays, RNA pull-down assays, and in vitro studies were employed to elucidate the anti-angiogenic mechanism of tRNA-Cys-5-0007. Exosomal formulation was employed to enhance the synergistic anti-angiogenic and anti-inflammatory efficacy of tRNA-Cys-5-0007.

**Results:**

tRNA-Cys-5-0007 expression was down-regulated under angiogenic conditions. Conversely, tRNA-Cys-5-0007 overexpression exhibited anti-angiogenic effects in retinal endothelial cells, as evidenced by reduced proliferation, sprouting, migration, and tube formation abilities. In diabetic, laser-induced CNV, and OIR models, tRNA-Cys-5-0007 overexpression led to decreased ocular vessel leakage, inhibited angiogenesis, and reduced ocular inflammation. Mechanistically, these effects were attributed to the targeting of vascular endothelial growth factor A (VEGFA) and TGF-β1 by tRNA-Cys-5-0007. The utilization of an exosomal formulation further potentiated the synergistic anti-angiogenic and anti-inflammatory efficacy of tRNA-Cys-5-0007.

**Conclusions:**

Concurrent targeting of tRNA-Cys-5-0007 for anti-angiogenic and anti-inflammatory therapy holds promise for enhancing the effectiveness of current anti-angiogenic therapy.

**Supplementary Information:**

The online version contains supplementary material available at 10.1186/s12967-024-05338-w.

## Introduction

Pathological angiogenesis is a hallmark feature in the pathogenesis of various human diseases, such as cancers, inflammatory disorders, and ocular diseases [1]. The eye offers a unique opportunity to non-invasively access the morphological and functional changes in blood vessels [2]. Ocular angiogenesis is a major cause of severe vision loss, which can occur in a spectrum of ocular diseases, such as retinopathy of prematurity (ROP), diabetic retinopathy (DR), and neovascular age-related macular degeneration (nAMD) [1]. Vascular endothelial growth factor (VEGF) is considered as the most important regulator of ocular angiogenesis. Increased VEGF expression causes the breakdown of blood-retinal barrier and ocular angiogenesis [3, 4]. Anti-VEGF therapy has shown as an effective method for treating ocular neovascular diseases [5, 6]. However, a substantial number of patients do not respond to anti-VEGF therapy [7]. Moreover, long-term anti-VEGF therapy can lead to the occurrence of subretinal fibrosis [8, 9]. Hence, there is a pressing need to deepen our understanding of the mechanisms underlying ocular angiogenesis and explore novel anti-angiogenic factors.

The major mechanisms underlying pathological angiogenesis involve tissue hypoxia, oxidative stress, and inflammatory response [10, 11]. Recent evidence has revealed that inflammation and angiogenesis are closely interconnected. Inflammation is shown as a critical driver of pathological angiogenesis. Monocytes/macrophages and microglia accumulate within the ischemic tissues, contributing to the progression of inflammatory response via the release of pro-inflammatory factors [12, 13]. These inflammatory factors accelerate blood-retinal barrier breakdown, vessel injury, and pathological angiogenesis. In turn, these neo-formed vessels aggravate inflammatory response by facilitating the transport of inflammatory cells, nutrients, and oxygen [12, 14, 15]. Thus, the concurrent use of anti-angiogenic and anti-inflammatory therapeutic modalities holds great promise for enhancing the treatment outcomes of ocular vascular diseases.

Epigenetic regulation refers to the control of gene expression without altering DNA sequences [16]. Recently, small RNAs-mediated epigenetic regulation has aroused great interests due to their roles in the pathogenesis of neovascular diseases [17]. Small RNAs can post-transcriptionally regulate gene expression by pairing with the target mRNAs through base complementarity via inhibiting mRNA translation and/or inducing mRNA degradation ^[15]^. Small RNAs include microRNAs (miRNAs), tRNA-derived small RNA (tsRNAs), piwi-interacting RNAs (piRNAs), and small nucleolar RNAs (snoRNAs) [18]. Unlike microRNAs, tsRNAs are not transcribed from the transcriptome DNAs, which are generated through the cleavage of tRNAs by the specific ribonucleases. tsRNA play versatile roles in a number of biological processes, such as gene silencing, RNA stability, gene translation, cell cycle, and apoptosis [19] Abnormal tsRNA expression is tightly associated with the occurrence and progression of human diseases. Notably, tsRNAs can play their roles via multi-target regulatory manners [20, 21]. If the target genes show similar affinity for a common tsRNA, these genes may respond synchronously and is thought to require fine-tuning of target affinities. In addition, tsRNA-mediated post-transcriptional regulation is more efficient in coordinating gene expression compared with transcriptional regulation [22].

However, as a novel type of small RNA, tsRNA therapy faces a significant hurdle in its rapid degradation owing to the high ribonuclease activities present in plasma. Clearance mechanisms such as bile excretion, renal filtration, and immunologic phagocytosis further compound this challenge. To address these issues, various delivery vehicles have been devised, encompassing viral vectors, liposomes, innovative synthetic materials, and biocarriers [23]. Exosomes are small vesicles with a single membrane ranging from 30 to 150 nm in diameter. They have intrinsic ability to traverse the barriers and transport small RNAs between cells [24, 25]. As therapeutic delivery agents, exosomes are tolerated by immune system because they are natural nanocarriers derived from endogenous cells [26]. Furthermore, exosomal formulation of therapeutic preparation was conducted to improve its therapeutic efficacy in addressing challenges such as inefficiency, non-specificity, and immunogenic reaction. Exosomes derived from genetically engineered cells can deliver small RNAs to target tissues and cells, specific surface ligands present on exosomes can attach to target cells, enabling the delivery of RNAs, proteins, or cytokines to stimulate specific biological functions within the target cells [27–29].

Through our prior sequencing analyses and bioinformatics predictions, tRNA-Cys-5-0007 emerged as a potential regulator of ocular angiogenesis. In this study, our focus was on investigating the anti-angiogenic and anti-inflammatory effects of tRNA-Cys-5-0007 in the context of ocular angiogenesis. tRNA-Cys-5-0007 exhibited significantly reduced expression levels across experimental angiogenesis models, including the STZ-induced diabetic model, laser-induced CNV model, and OIR model, indicating consistent down-regulation in pathological ocular angiogenesis. Overexpression of tRNA-Cys-5-0007 demonstrated anti-angiogenic effects by attenuating endothelial cell proliferation, sprouting, migration, and tube formation capabilities, ultimately resulting in diminished ocular vascular leakage, angiogenesis, and inflammation. Mechanistically, tRNA-Cys-5-0007 suppressed ocular angiogenesis and inflammation through the regulation of VEGFA and TGF-β1 expression. Reduced VEGFA expression inhibited pathological angiogenesis by suppressing endothelial cell proliferation, migration, tube formation, and sprouting. Concurrently, inhibition of TGF-β1 expression alleviated ocular vascular leakage, angiogenesis, and leukocyte infiltration in the retina and choroid. Moreover, the exosomal formulation of tRNA-Cys-5-0007 amplified its synergistic anti-angiogenic and anti-inflammatory efficacy, underscoring the potential of tRNA-Cys-5-0007 as a promising therapeutic target for ocular neovascular diseases.

## Materials and methods

### Animal experiment

C57BL/6J mice were obtained from the Model Animal Research Center of Nanjing University (Nanjing, China). The mice were housed in at 25 °C with a 12:12 h light-dark cycle under specific-pathogen free conditions and provided with ad libitum access to water and fed standard laboratory chow.

### Cell culture

Human retinal vascular endothelial cells (HRVECs, ACBRI-181, Cell Systems, USA) were cultured in the Endothelial Cell Medium (ECM, 1001, ScienCell, USA) supplemented with 5% fetal bovine serum (FBS, SC-0025, ScienCell, USA), 1% endothelial cell growth supplement (ECGS, SC-1052, ScienCell, USA), and 1% penicillin/streptomycin (PS, SC-0503, ScienCell, USA). They were maintained at 37 °C under the humidified condition with 5% CO_2_. When the cells reached about 75% density, they were transfected using Lipofectamine 3000 Reagent (L3000015, Thermo Fisher, USA). Human umbilical-cord-derived mesenchymal stem cells (hUC-MSCs, RC01005, Nuwacell, China) were cultured in Nuwacell® ncMission hMSC Medium (RP02010, Nuwacell, China) at 37 °C under the humidified condition with 5% CO_2_.

### Exosome isolation

Cell culture medium was subjected for sequential centrifugation at 1000 × *g* for 10 min, followed by 10,000 × *g* for 30 min. The resulting supernatant was collected and filtered using a 0.22 μm filter (SLGP033RB, Millex, Germany), followed by ultracentrifugation at 100,000 × *g* for 1 h. Exosome pellets were then washed with PBS, recovered by centrifugation at 100,000 × *g* for 1 h and re-suspended in sterile PBS for the subsequent experiment. The protein concentration of isolated exosomes was quantified by the Bradford assay kit (C503041, Sangon Biotech, China).

### Exosome characterization

The morphology of purified exosomes was visualized by transmission electron microscopy (TEM, FEI, Tecnai G2, USA). The concentration and size distribution of exosome was determined by the Nanoparticle Tracking Analysis (NTA) technology with ZetaView PMX 110 (Particle Metrix, Meerbusch, Germany). Western blot analysis was conducted to detect the expression of exosome-associated markers. Exosomal particles and cultured cells were lysed in RIPA lysis buffer (P0013B, Beyotime, China) containing complete™ Protease Inhibitor Cocktail (04693116001, Roche, Switzerland). The protein concentration was determined and normalized by the BCA method (23,225, Thermo Fisher, USA). Protein samples were prepared with a five-fold loading buffer and subjected to SDS-polyacrylamide gel electrophoresis (SDS-PAGE) for protein separation. The separated proteins were transferred to a PVDF membrane (IPVH00010, Merck Millipore, Germany). The expression of exosomal surface markers, including CD81 (1:1000, sc-166,029, Santa Cruz, USA), CD63 (1:1000, sc-5275, Santa Cruz, USA), Tsg101 (1:1000, sc-7964, Santa Cruz, USA), and calnexin (1:1000, sc-23,954, Santa Cruz, USA), was detected.

### Preparation of exosomal formulation of tRNA-Cys-5-0007

Exosomes were loaded with tRNA-Cys-5-0007 mimics or negative control (NC) mimics using the CUY21EDIT II electroporation system (BEX, Japan). The electroporation mixture was prepared by combining exosomes and mimics in a 1:1 (wt/wt) ratio in the electroporation buffer, resulting in a final concentration of 1 mg/mL for exosomes in the mixture. To perform electroporation, 200 µL of the mixture was transferred to ice-cold BEX cuvettes (SE-202, BEX, Japan) and subjected to the following parameters using the electroporation system: Pp V: 80 V, Pd V: 25 V, Pp on: 6.00 ms, Pd on: 50.0 ms, Pp off: 10.0 ms, Pd off: 50.0 ms, Pd Cycle N: 10, Capa: 940 µF. Following the electroporation, the mixture was transferred to a new Eppendorf tube and incubated at 37 °C for 30 min. The unloaded mimics were removed using the ultracentrifugation. The formulated exosomes were re-suspended in DPBS (D8537, Sigma, USA) and stored at -80 °C until further usage.

### STZ-induced diabetic model

Diabetes mellitus was induced in C57BL/6 mice (10–12 weeks old) via intraperitoneal injections of streptozotocin (STZ, 2196GR001, BioFroxx, German) after fasting for 12 h. Intraperitoneal injection of STZ solution was performed using 1 mL syringes and 25 G needles at a concentration of 40 mg/kg of body weight. The control group received an intraperitoneal injection of vehicle (citrate buffer). The blood glucose level was measured at 7 d after STZ injection and monitored weekly thereafter. Diabetes mellitus was confirmed by the random blood glucose level more than 300 mg/dl [30].

#### Laser-induced choroidal neovascularization model

C57BL/6J mice (6–8 weeks old) were anesthetized and their pupils were dilated by 0.5% tropicamide with 5% phenylephrine. Ofloxacin eye ointment was applied to the eye’s surface. Laser photocoagulation was performed around optic nerve using the OcuLight GLx Laser System (Iridex, USA). The following parameters were selected: a wavelength of 532 nm, a spot size of 50 μm, a duration of 70 ms, and a power of 140 mW. The appearance of bubbles indicated the disruption of Bruch’s membrane, confirming successful modeling. Only laser spots that resulted in visible formation of vaporization bubbles were included in the study [31].

### Oxygen-induced retinopathy (OIR) model

The murine model of OIR was used to reproduce retinopathy of prematurity (ROP) and investigate pathologic neovascularization in the retina. Briefly, OIR model was induced in C57BL/6J mice. The neonatal mice and their nursing mother were exposed to hyperoxic conditions (75% O_2_) from the postnatal day 7 (P7) to P12, causing vaso-obliteration (hyperoxia phase). Subsequently, these mice were returned to room air (21% O_2_) until P17, causing hypoxia-driven revascularization and pathologic neovascularization (hypoxic-ischemic phase). The age-matched controls were reared simultaneously in room air [32].

### Intravitreal injection

The mice were anesthetized using 1.25% 2, 2, 2-tribromoethanol (0.2 ml/10 g, M2910, Nanjing Aibei Biotechnology, China), one drop of 0.5% tropicamide with 5% phenylephrine was used to dilate the pupils. Corneal anesthesia was performed using 0.4% oxybuprocaine (Benoxil, Santen, Japan). The eyeball was fully exposed under a stereo surgical microscope (SZ61, Olympus, Japan). A 30-gauge (30 G) needle was used to poke a small hole at 1 mm behind corneoscleral limbus avoiding the vascular network. Then, a 33 G needle (Hamilton) was inserted to inject the reagent into vitreous chamber. In DR model, injections were conducted once a week after establishing diabetes for 3 months. The retinas were collected for analysis 4 months after the onset of diabetes. In OIR model, injections were performed on P12 and the retinas were harvested on P17. In CNV model, injections were administered immediately following laser injury and the choroid was harvested at day 14 following laser injury.

### Statistical analysis

Data analysis was carried out using one-way analysis of variance (ANOVA) followed by Tukey’s multiple comparisons test and unpaired two-tailed Student’s *t* test by Graphpad prism 8 software. Homogeneity test of variances and normal distributions were determined before each test. All data were expressed as mean ± SD and *P* < 0.05 considered statistically significant.

Detailed experimental methods can be found in the supplementary materials.

## Results

### tRNA-Cys-5-0007 expression is reduced during ocular angiogenesis

**Fig. 1:**
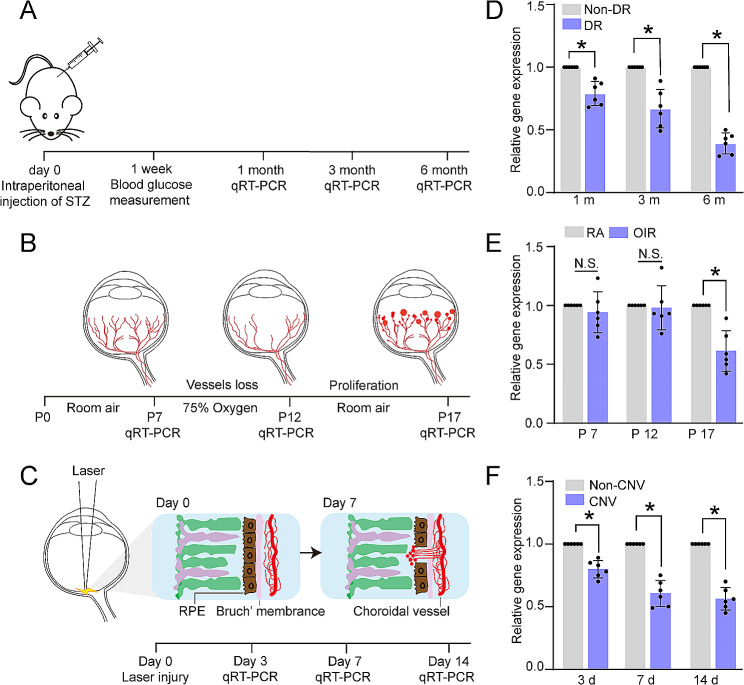
tRNA-Cys-5-0007 expression is reduced during ocular angiogenesis. (**A**-**C**) Schematic diagram depicting the experimental design of murine model of DR (**A**), OIR (**B**), and CNV (**C**). (**D**) qRT-PCR assays were performed to detect the expression of tRNA-Cys-5-0007 in DR model at 1 m, 3 m, and 6 m after STZ injection. (**E**) qRT-PCR assays were performed to detect the expression levels of tRNA-Cys-5-0007 in the murine model of OIR at P7, P12, and P17. (**F**) qRT-PCR assays were performed to detect the levels of tRNA-Cys-5-0007 expression in the RPE/choroid complex of C57BL/6J mice on day 3, 7, and 14 after laser photocoagulation. The data were presented as the means ± SD. *n* = 6 per group; *P < 0.05; Student’s *t* test

To interrogate the involvement of tRNA-Cys-5-0007 in ocular angiogenesis, we determined its expression pattern in the murine models of DR, OIR, and CNV, which have been widely used to investigate ocular angiogenesis (Fig. 1A-C). STZ-induced diabetic model is a popular model for DR research [33, 34]. qRT-PCR assays revealed that the levels of tRNA-Cys-5-0007 expression were significantly decreased in diabetic retinas compared with the non-diabetic controls at 1, 3, and 6 months following diabetes induction (Fig. 1D). OIR model has been employed to reproduce retinopathy of prematurity (ROP) [33, 34]. qRT-PCR assays showed that the levels of tRNA-Cys-5-0007 expression remained unchanged at P7 and P12 compared to room air (RA) group. However, the levels of tRNA-Cys-5-0007 expression were significantly decreased at P17 compared to RA group (Fig. 1E). CNV was induced in C57BL/6 mice by laser photocoagulation, which can cause the destruction of Bruch’s membranes. This model has been utilized for studying subretinal neovascularization in neovascular age-related macular degeneration (nAMD) [33, 34]. qRT-PCR assays revealed that the levels of tRNA-Cys-5-0007 expression in the RPE/choroids of CNV mice were lower than that in the normal controls at day 3, day 7 and day 14 following laser injury (Fig. 1F). HRVECs were exposed to high glucose or VEGF condition to mimic angiogenic condition in vitro. The levels of tRNA-Cys-5-0007 expression displayed a gradual decrease at 24 h, 48 h, and 72 h following high glucose stress (Fig. S1A). Additionally, the levels of tRNA-Cys-5-0007 expression were reduced following VEGF treatment (Fig. S1B). These results reveal a significant correlation between tRNA-Cys-5-0007 and the development of ocular angiogenesis.

### tRNA-Cys-5-0007 regulates ocular angiogenesis and inflammation in vivo

**Fig. 2:**
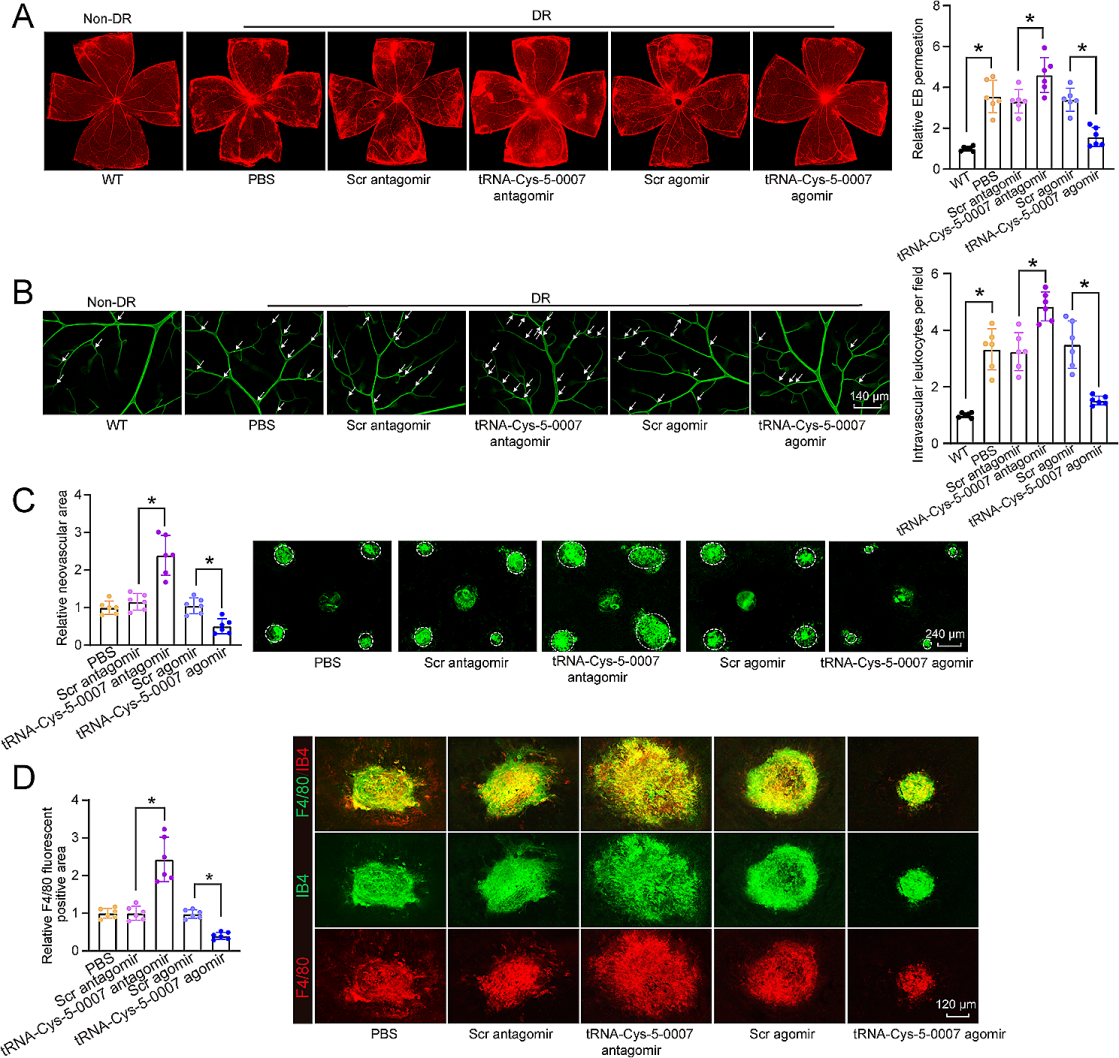
tRNA-Cys-5-0007 overexpression plays an anti-angiogenic and anti-inflammatory role during ocular angiogenesis. (**A** and **B**) Diabetic C57BL/6J mice (8-week-old, male) received intravitreous injections of PBS, scramble (Scr) agomir, tRNA-Cys-5-0007 agomir, Scr antagomir, tRNA-Cys-5-0007 antagomir. Wild-type C57BL/6J mice (WT) were taken as the controls. The mice were perfused with Evans blue dye for 30 min. The fluorescence signal of flat-mounted retina was detected by a fluorescence microscope. The representative images were shown. The infiltrated leukocytes in retinal vessels were detected by fluorescein-isothiocyanate (FITC)-coupled concanavalin A lectin perfusion assays. Adherent leukocytes (white arrows) were stained with FITC-conA (**B**). (**C** and **D**) CNV mice (8-week-old, male) received intravitreous injections of PBS, Scr agomir, tRNA-Cys-5-0007 agomir, Scr antagomir, tRNA-Cys-5-0007 antagomir, or left untreated. The RPE/choroid complexes were collected at day 14 and stained with IB-4 to label CNV lesions. The representative images were shown. Green staining indicated CNV lesions; dashed lines indicated CNV areas (**C**). On day 14 following laser injury, CNV lesions were stained with IB-4 (green) and F4/80 (red) to label CNV lesions and macrophages (**D**). The data were presented as the means ± SD. *n* = 6 mice per group; *P < 0.05; One-way ANOVA followed by Tukey’s multiple comparisons test

Due to altered tRNA-Cys-5-0007 expression during pathological angiogenesis, we speculated that tRNA-Cys-5-0007 was a potential regulator of pathological angiogenesis. tRNA-Cys-5-0007 antagomir (sequence: 5’-UACCCCUGAGCUAUACCCCC-3’) was designed to reduce the expression levels of tRNA-Cys-5-0007. Conversely, tRNA-Cys-5-0007 agomir (sequence: 5’-GGGGGUAUAGCUCAGGGGUA-3’) was designed to enhance the expression levels of tRNA-Cys-5-0007. qRT-PCR assays showed that intravitreal injection of tRNA-Cys-5-0007 agomir significantly elevated tRNA-Cys-5-0007 levels in the retinas (Fig. S2A). In DR model, Evans blue assays were conducted to detect blood-retina barrier (BRB) disruption. The result showed that intravitreal injection of tRNA-Cys-5-0007 antagomir aggravated diabetes-induced retinal vascular permeability compared with STZ-induced diabetic mice. By contrast, intravitreal injection of tRNA-Cys-5-0007 agomir had an opposite effect on retinal vascular permeability, showing reduced retinal vascular leakage (Fig. 2A). Claudin5 is a tight junction component, a well-known determinant of blood vessel barrier integrity [35]. Double-fluorescent staining of claudin5/IB4 was conducted to detect the integrity of blood-retina barrier. The result showed that intravitreal injection of tRNA-Cys-5-0007 antagomir disrupted blood-retina integrity, as shown by decreased areas of claudin5/IB4 overlapped regions. By contrast, injection of tRNA-Cys-5-0007 agomir increased the areas of claudin5/IB4 overlapped regions (Fig. S2B). Diabetes can cause capillary leukocytosis and capillary non-perfusion in the retinas [36]. To determine the role of tRNA-Cys-5-0007 in vascular inflammation, retinal leukocytosis assays were performed. The results showed injections of tRNA-Cys-5-0007 antagomir significantly increased leukocyte infiltration in diabetic retinas compared to the non-diabetic controls. By contrast, injection with tRNA-Cys-5-0007 agomir reduced the number of adherent leukocytes in retinal capillaries (Fig. 2B).

Laser-induced CNV model was established to determine the role of tRNA-Cys-5-0007 in choroidal angiogenesis. CNV lesions were labeled by Isolectin GS-IB4 staining. Intravitreal injection of tRNA-Cys-5-0007 antagomir increased the areas of laser-induced CNV regions, while intravitreal injection of tRNA-Cys-5-0007 agomir effectively decreased the areas of CNV regions (Fig. 2C). Macrophages serve as the major inflammatory infiltrating cells in CNV lesions [37]. F4/80 is a specific marker of macrophages [38]. We conducted F4/80/IB4 double staining assays and F4/80/IB4 overlapped areas were determined. The results showed that intravitreal injection of tRNA-Cys-5-0007 antagomir increased F4/80/IB4 overlapped areas, while intravitreal injection of tRNA-Cys-5-0007 agomir alleviated inflammatory cell infiltration as shown by smaller F4/80/IB4 overlapped areas (Fig. 2D). OIR model was employed to investigate the role of tRNA-Cys-5-0007 in retinal neovascularization in vivo. Injection of tRNA-Cys-5-0007 antagomir exacerbated pathological retinal neovascularization and increased avascular areas at P17 in OIR retinas compared with scramble antagomir group (Fig. S2D-2 F). Conversely, injection of tRNA-Cys-5-0007 agomir led to a reduction in both the angiogenic areas and avascular areas when compared with scramble agomir (Fig. S2D-2 F).

### tRNA-Cys-5-0007 regulates endothelial cell function in vitro

**Fig. 3:**
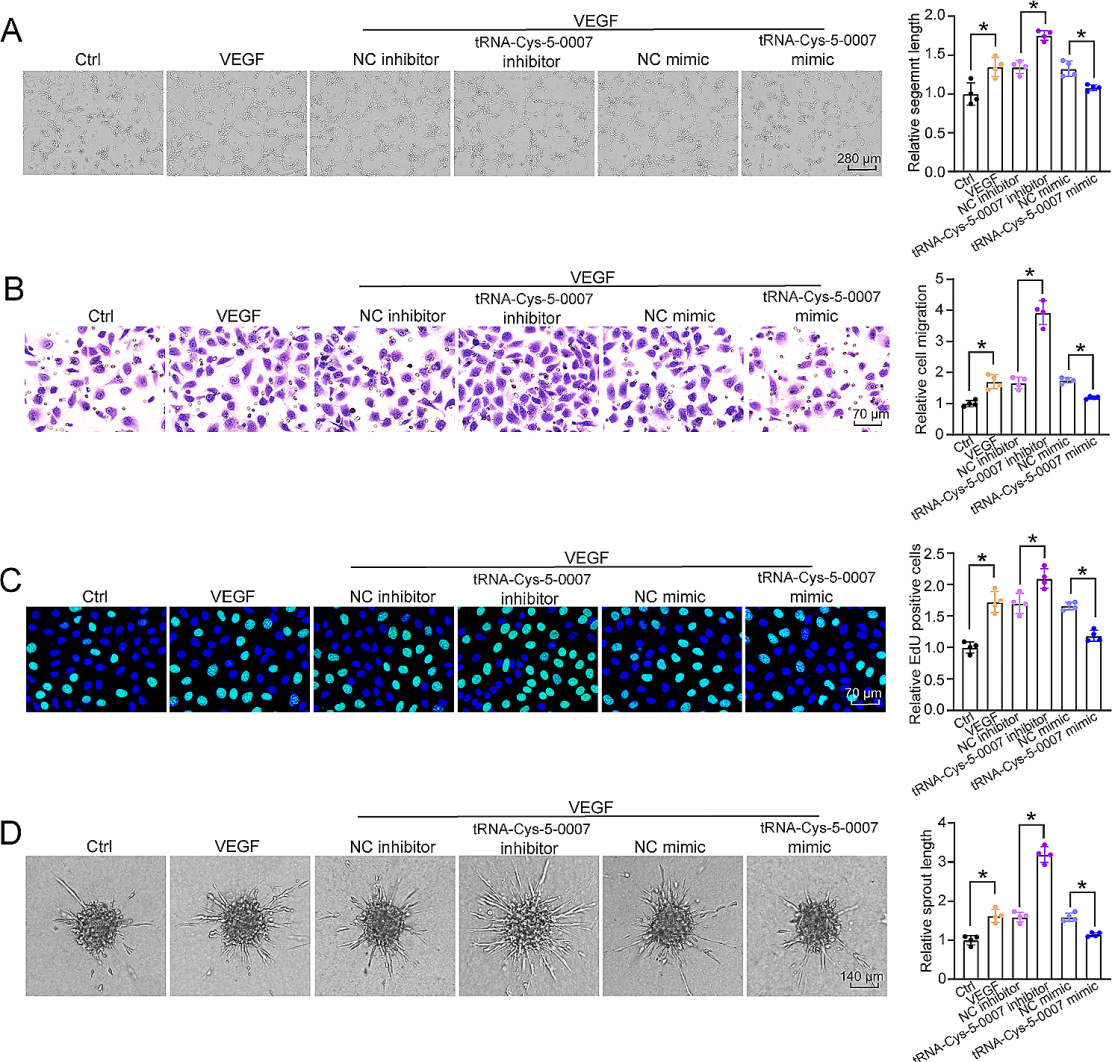
tRNA-Cys-5-0007 regulates endothelial angiogenic function. (**A**-**D**) HRVECs were transfected with negative control (NC) inhibitors, tRNA-Cys-5-0007 inhibitors, NC mimics, tRNA-Cys-5-0007 mimics, or left untreated (Ctrl) for 24 h. Representative images and quantification results of tube formation assays were shown (**A**). Representative images and quantification results of transwell migration assays were shown (**B**). Representative images and quantification of EdU proliferation assays were shown (**C**). Representative images and quantification results of endothelial cell spheroid‑based sprouting assays were shown (**D**). The data were presented as the means ± SD. *n* = 4 per group; *P < 0.05; One-way ANOVA followed by Tukey’s multiple comparisons test

Endothelial cells (ECs) line the intimal layer of vascular system, playing a critical role upon pathological stimuli to maintain vascular homeostasis [39]. Human retinal vascular endothelial cells (HRVECs) were used to investigate the role of tRNA-Cys-5-0007 in vitro. tRNA-Cys-5-0007 mimics (sequence: 5’-GGGGGUAUAGCUCAGGGGUA-3’) and tRNA-Cys-5-0007 inhibitors (sequence: 5’-UACCCCUGAGCUAUACCCCC-3’) were separately transfected into HRVECs. Transfection of tRNA-Cys-5-0007 mimics led to increased tRNA-Cys-5-0007 levels (Fig. S3A). tRNA-Cys-5-0007 inhibitor was also successfully transfected into HRVECs (Fig. S3B). The angiogenic effects were determined by tube-formation assays, transwell migration assays, 5-ethynyl-2’-deoxyuridine (EdU) assays, and spheroid-based sprouting assays. Transfection of tRNA-Cys-5-0007 mimics suppressed the tube formation capacity as shown as less tube length, while transfection of tRNA-Cys-5-0007 inhibitors promoted tube formation capacity in HRVECs (Fig. 3A). Transwell migration assays were used to evaluate cell migratory ability. The results showed that transfection of tRNA-Cys-5-0007 mimics reduced the number of migrated cells, while transfection of tRNA-Cys-5-0007 inhibitors increased the effect of HRVECs migration ability (Fig. 3B). EdU assays demonstrated that transfection of tRNA-Cys-5-0007 mimics reduced the proliferation ability of HRVECs, while downregulation of tRNA-Cys-5-0007 levels by inhibitors significantly boosted EdU positive cells of HRVECs (Fig. 3C). We further performed the spheroid-based sprouting assays to determine endothelial sprouting ability. The results showed that transfection of tRNA-Cys-5-0007 mimics reduced the sprouting length of endothelial cells compared with the control group, while transfection of tRNA-Cys-5-0007 inhibitors had the opposite effects (Fig. 3D).

### tRNA-Cys-5-0007 regulates endothelial angiogenic effects by targeting VEGFA and TGF-β1

**Fig. 4:**
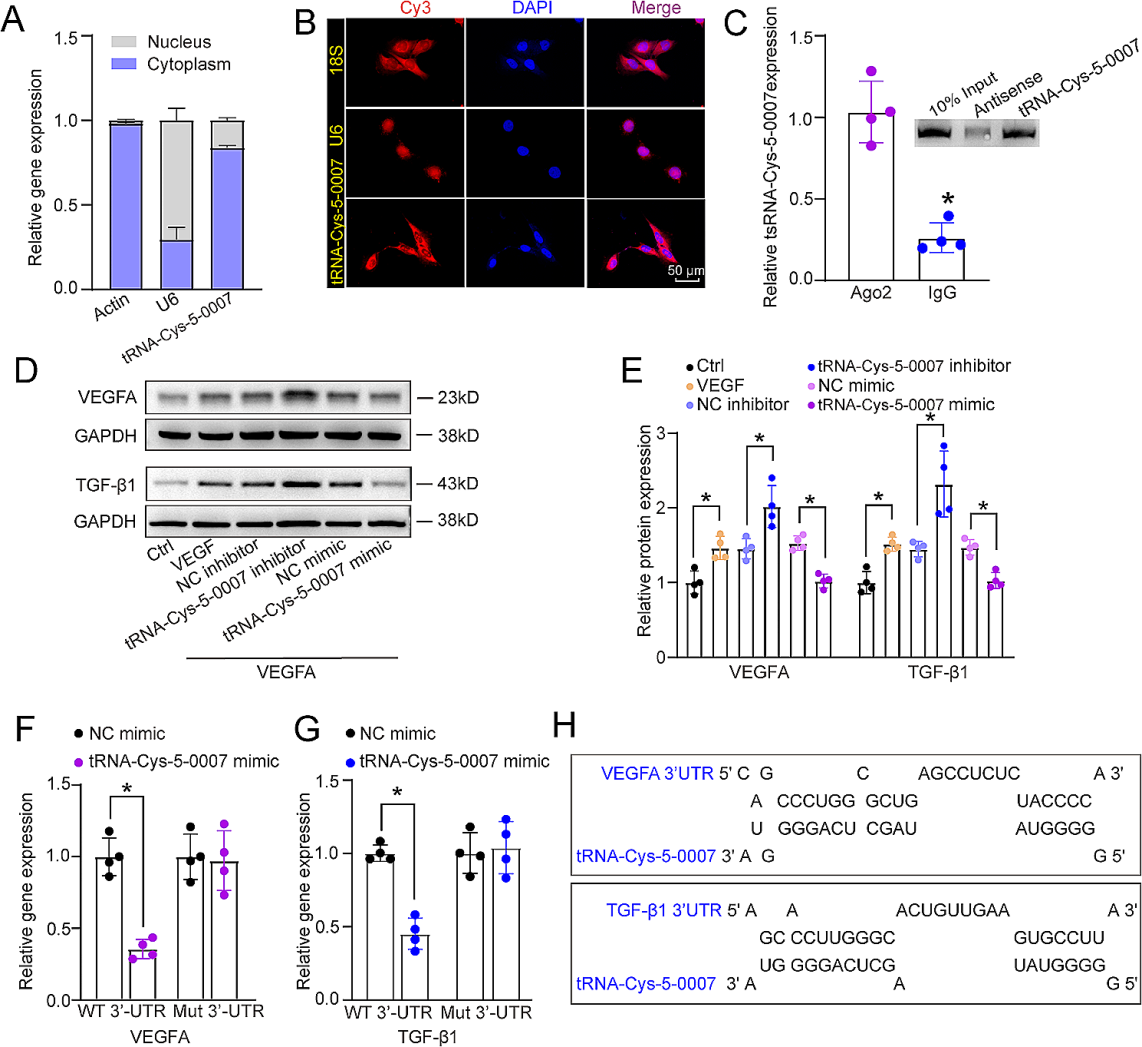
tRNA-Cys-5-0007 regulates endothelial angiogenic effects by targeting VEGFA and TGF-β1. (**A**) The expression distribution of U6, actin, and tRNA-Cys-5-0007 was examined by qRT-PCRs in the nucleus fractions and cytoplasm fractions of HRVECs. (**B**) FISH assays were conducted to detect the cellular distribution of tRNA-Cys-5-0007. 18 S rRNA and U6 were detected as the cytoplasm control and nucleus control. (**C**) The fractions of HRVECs were immunoprecipitated using Ago2 or IgG antibody. The amounts of tRNA-Cys-5-0007 in immunoprecipitates were examined by qRT-PCRs. The biotinylated tRNA-Cys-5-0007 or its anti-sense RNA was incubated, targeted with streptavidin beads, and washed. Western blots were then conducted to detect the specific interaction between Ago2 and tRNA-Cys-5-0007. (**D** and **E**) The levels of VEGFA and TGF-β1 expression were examined by western blots in HRVECs following the transfection of negative control (NC) inhibitors, tRNA-Cys-5-0007 inhibitors, NC mimics, tRNA-Cys-5-0007 mimics, incubated with or without VEGF (Ctrl) for 24 h. (**F**–**H**) The luciferase activity of wild-type VEGFA/TGF-β1 or mutant VEGFA/ TGF-β1 following the transfection with NC mimics or tRNA-Cys-5-0007 mimics for 24 h in HRVECs were detected. The data were presented as the means ± SD; *n* = 4 per group; *P < 0.05; Student’s *t* test

We next explored the molecular mechanism of tRNA-Cys-5-0007 in endothelial angiogenic effects. Nucleocytoplasmic separation analysis using qRT-PCR assays and fluorescence in situ hybridization (FISH) assays revealed that tRNA-Cys-5-0007 was mainly expressed in the cytoplasm of HRVECs (Fig. 4A and B). Argonaute 2 (Ago2) incorporates a multitude of microRNAs as guides, each targeting a diverse set of mRNAs. RNA immunoprecipitation (RIP) and RNA pull down assay demonstrated that tRNA-Cys-5-0007 could be immunoprecipitated by anti-Ago2 but not anti-immunoglobulin (Ig) G antibody (Fig. 4C). Due to direct binding between Ago2 and tRNA-Cys-5-0007, we speculated that tRNA-Cys-5-0007 played its role at the post-transcriptional level through a miRNA-like mechanism. The potential target genes of tRNA-Cys-5-0007 were predicted by the tsRTar database (http://www.rnanut.net/tRFTar/). To further annotate the target genes, we performed pathway analysis of target genes using the Wikipathways (https://www.wikipathways.org/). The results demonstrated that the target genes of tRNA-Cys-5-0007 were potentially involved in ocular angiogenesis and inflammation-related pathways, such as VEGFA-VEGFR signaling pathway, TGF-β signaling pathway, and IL-18 signaling pathway (Fig. S4A). Meanwhile, VEGFA and TGF-β1 were predicted as the potential target genes using the tsRFun database (https://rna.sysu.edu.cn/tsRFun/). We choose VEGFA and TGF-β1 as the potential targets of tRNA-Cys-5-0007 for further study. TGF-β1, the prototypical member of TGF-β family, consists of three isoforms in mammals (TGF-β1, β2, and β3), among which TGF-β1 is the most abundant and well-studied family member. Western blots showed that the levels of VEGFA and TGF-β1 expression were reduced following the transfection of tRNA-Cys-5-0007 mimics in HRVECs. By contrast, transfection of tRNA-Cys-5-0007 inhibitors led to increased levels of VEGFA and TGF-β1 expression (Fig. 4D and E). Similar expression trend was detected in ELISA assays (Fig. S4B). Furthermore, we used the luciferase assays to verify the direct binding between tRNA-Cys-5-0007 and VEGFA or TGF-β1. Transfection of tRNA-Cys-5-0007 mimics caused a marked reduction in luciferase activity when wild-type 3’-UTR (untranslated region) of VEGFA and TGF-β1 was present (Fig. 4F–H). However, there was no change of luciferase activity of mutant 3’-UTR of VEGFA and TGF-β1 following the transfection of tRNA-Cys-5-0007 mimics (Fig. 4F–H).

### Exosomal preparation and optimization of tRNA-Cys-5-0007 loading in exosomes

The above-mentioned results show that tRNA-Cys-5-0007 is a promising nucleic acid drug for ocular angiogenesis. However, small RNA-based therapies have limitations due to lacking an effective delivery system. Recently, exosomes have attracted great attentions for the next-generation drug delivery system due to low immunogenicity, high biocompatible structure, robust membrane avoiding nucleic acid degradation, and well-tolerated in body fluids.

**Fig. 5:**
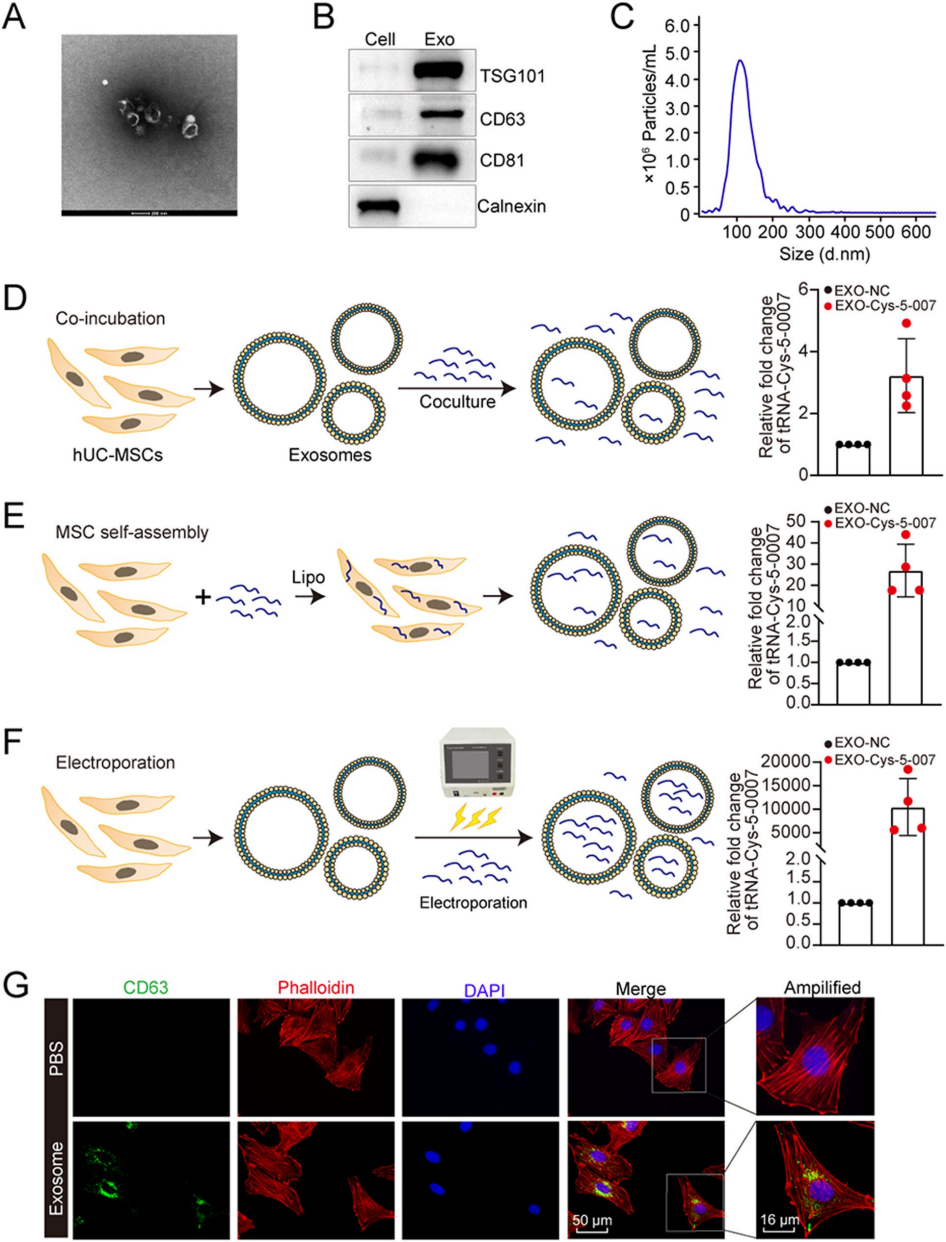
Exosomal preparation and optimization of tRNA-Cys-5-0007 loading in exosomes. (**A**) The morphology of purified exosomes from hUC-MSCs were observed by TEM. (**B**) Western blot analysis of exosome surface markers (TSG101, CD63, and CD81) and the negative exosome marker (calnexin) in hUC-MSCs and hUC-MSCs-Exos. (**C**) Diameter of hUC-MSCs-Exos were analyzed by the Nanoparticle tracking analysis (NTA) technology using ZetaView PMX 110. (**D**-**F**) Three different approaches (co-incubation, self-assembly, and electroporation) were used to load the negative control (NC) mimics or tRNA-Cys-5-0007 mimics into exosomes. The loading efficiency was detected by qRT-PCR assays. (**G**) Uptake of hUC-MSCs-derived exosomes by HRVECs. The cell nuclei were stained with DAPI (blue) and cytoskeletons were stained with Phalloidin (red). The data were presented as the means ± SD; *n* = 4 per group; *P < 0.05; Student’s *t* test

To investigate whether exosomes can act as an ideal cargo carrier to deliver tRNA-Cys-5-0007 to endothelial cells, the hUC-MSCs-derived exosomes were isolated and purified by differential centrifugation. Transmission electron microscopy (TEM) analysis revealed the presence of cup-shaped structures for hUC-MSCs-derived exosomes, which is a classical feature of exosomes (Fig. 5A). Western blot assays demonstrated that the isolated exosomes expressed high levels of exosome-associated markers, such as CD81, CD63, and TSG101, in comparison to the whole cell lysate of hUC-MSCs. Notably, the negative exosomal marker, Calnexin, was not detected, indicating the purity of the isolated exosomes (Fig. 5B). Nanoparticle tracking analysis (NTA) was performed to determine the size distribution of exosomes. NTA analysis revealed a peak diameter of 115.1 nm, which was within typical size range of exosomes (Fig. 5C). We employed three different approaches: co-incubation, self-assembly, and electroporation, for loading scramble mimics or tRNA-Cys-5-0007 mimics into the exosomes. We performed qRT-PCR assays to detect the levels of tRNA-Cys-5-0007 expression in the exosomes. The results demonstrated that electroporation caused the highest loading efficiency of tRNA-Cys-5-0007 mimics into the exosomes (Fig. 5D-F).

We further characterized Exos-Cys-5-0007 using TEM, NTA, and western blots. TEM analysis revealed that Exos-Cys-5-0007 displayed the characteristic cup-like shape observed in exosomes. NTA measurements indicated that the mean size of Exos-Cys-5-0007 was 133.6 nm in the diameter. Western blot analysis demonstrated the presence of exosome-specific marker, TSG101, in both exosomes and Exos-Cys-5-0007 (Fig. S5A). To investigate the uptake of exosomes in vivo, we performed intravitreal injection of Evlink-labeled exosomes and isolated the choroids at day 3 following injection. Fluorescence microscopy revealed that Evlink-labeled exosomes were aggregated in CNV lesions (Fig. S5B). To determine the delivery efficiency, mice bioluminescence imaging was performed using in IVIS, Cys-labeled tsRNA-Cys-5-0007 or Exos-Cys-5-0007 were intravitreally injected. Fluorescence of the eye was imaged at 24 h after the injection. Exos-Cys-5-0007 enhanced retention effects of tsRNA-Cys-5-0007 (Fig. S5C). Additionally, we labeled the exosomes with CD63 and labeled the cytoskeleton with phalloidin. Effective uptake of exosomes was observed in HRVECs (Fig. 5G). Furthermore, fluorescence staining revealed the co-localization between CD63 and phalloidin in HRVECs (Fig. S5D).

### Safety evaluation of exosomal formulation of tRNA-Cys-5-0007 in vivo and in vitro

**Fig. 6:**
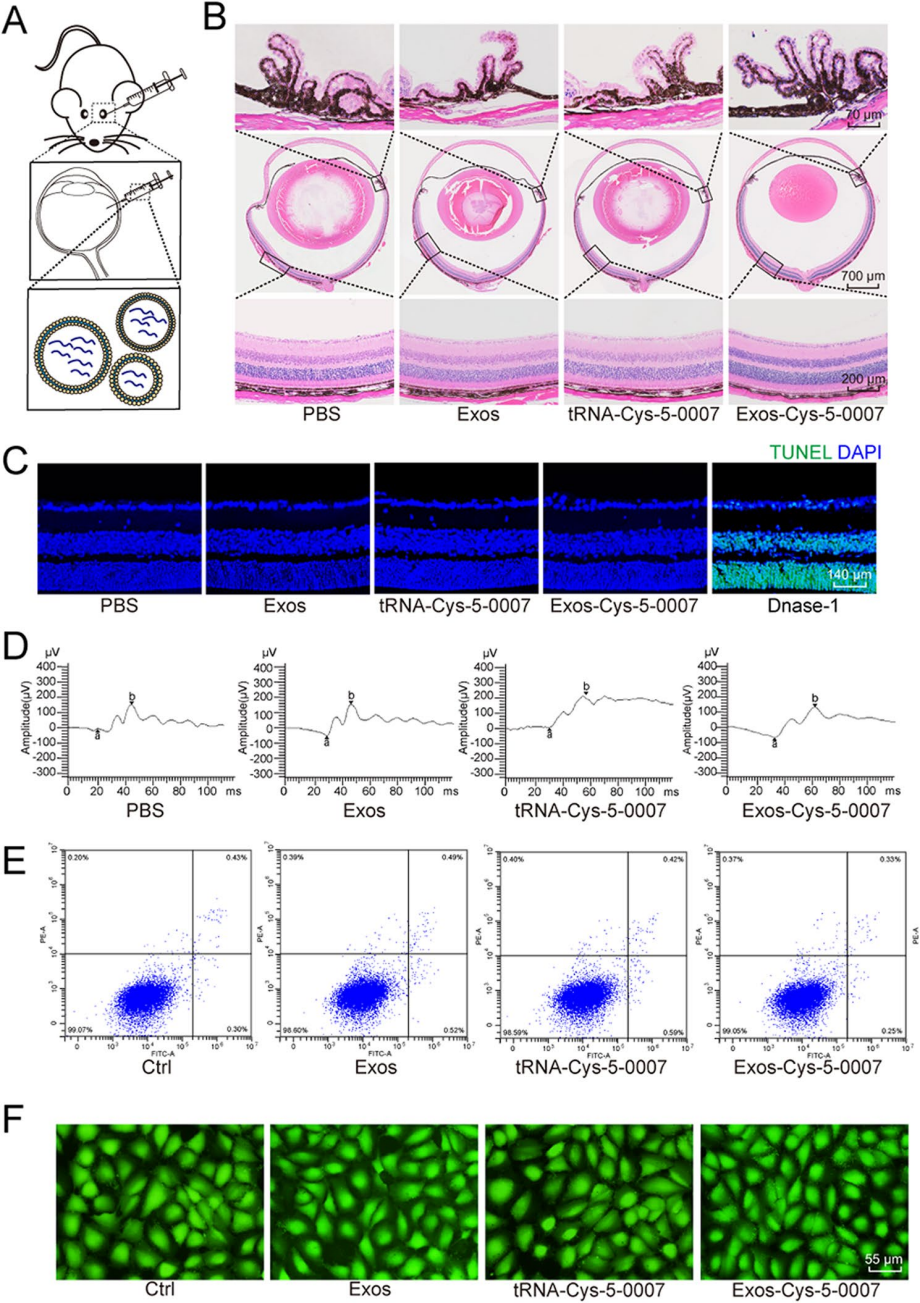
Safety evaluation of exosomal formulation of tRNA-Cys-5-0007 in vivo and in vitro. (**A**-**D**) The mice received intravitreal injections of PBS, exosomes, tRNA-Cys-5-0007, or Exos-Cys-5-0007 for 7 days. H&E staining was performed to detect the change of retinal structures. The representative images were shown (**B**, *n* = 5). TUNEL assays were conducted to detect retinal apoptosis along with the representative images (**C**, *n* = 5). ERG assays were conducted to detect the change of visual function (**D**, *n* = 5). (**E** and **F**) HRVECs were incubated with exosomes, tRNA-Cys-5-0007, Exos-Cys-5-0007, or PBS (Ctrl) for 24 h. Flow cytometry assays with Annexin V-FITC staining (**E**, *n* = 5) and PI/Calcein-AM double staining (**F**, *n* = 5) were conducted to detect the change of retinal apoptosis

To determine the safety of exosomes, tRNA-Cys-5-0007, and Exos-Cys-5-0007 as delivery vehicles in vivo, the mice received intravitreal injections of PBS, exosomes, tRNA-Cys-5-0007, and Exos-Cys-5-0007 for 7 days (Fig. 6A). Hematoxylin and eosin (H&E) staining was performed to determine the change of retinal structure. No obvious histopathological abnormalities or lesions were detected following the intravitreal injections of PBS, exosomes, tRNA-Cys-5-0007, and Exos-Cys-5-0007 (Fig. 6B). TUNEL assays were employed to detect retinal apoptosis. There was no significant increase in retinal apoptosis following the injections of exosomes, tRNA-Cys-5-0007, or Exos-Cys-5-0007 compared with PBS group (Fig. 6C). Electroretinography (ERG) assays showed that compared with PBS group, injections of exosomes, tRNA-Cys-5-0007, or Exos-Cys-5-0007 did not cause a marked change of a-wave or b-wave following the intravitreal injections of exosomes, tRNA-Cys-5-0007, and Exos-Cys-5-0007 (Fig. 6D).

To evaluate the potential systemic toxicity of exosomes, tRNA-Cys-5-0007, and Exos-Cys-5-0007, the comprehensive analyses including weight monitoring, hematologic analysis, and blood biochemistry were also performed at day 7 following intravitreal injections. Throughout the experimental period, there was no significant difference of body weight loss and blood glucose change among different experimental group (Fig. S6A and S6B). Compared with PBS group, intravitreal injections of exosomes, tRNA-Cys-5-0007, and Exos-Cys-5-0007 did not alter hematological parameters, including white blood cell (WBC) and red blood cell (RBC) counts (Fig. S6C and S6D). The indicators of liver function, such as alanine aminotransferase (ALT) and aspartate aminotransferase (AST), did not exhibit significant difference among the different experimental groups (Fig. S6E and S6F).

We also evaluated the potential cytotoxic effects of exosomes, tRNA-Cys-5-0007, and Exos-Cys-5-0007 in vitro. Flow cytometry and Calcein-AM/PI double staining assays were used to detect the apoptosis of HRVECs following the incubation with exosomes or Exos-Cys-5-0007 for 24 h. The results showed that incubation with exosomes or Exos-Cys-5-0007 did not induce a detected apoptosis in HRVECs (Fig. 6E and F).

### Exosomal formulation enhances the synergistic anti-angiogenic and anti-inflammatory efficacy of tRNA-Cys-5-0007

To determine whether exosomal formulation enhanced the synergistic anti-angiogenic and anti-inflammatory efficacy of tRNA-Cys-5-0007, we further employed DR, OIR and CNV models to assess the synergistic anti-angiogenic and anti-inflammatory efficacy of Exos-Cys-5-0007.

**Fig. 7:**
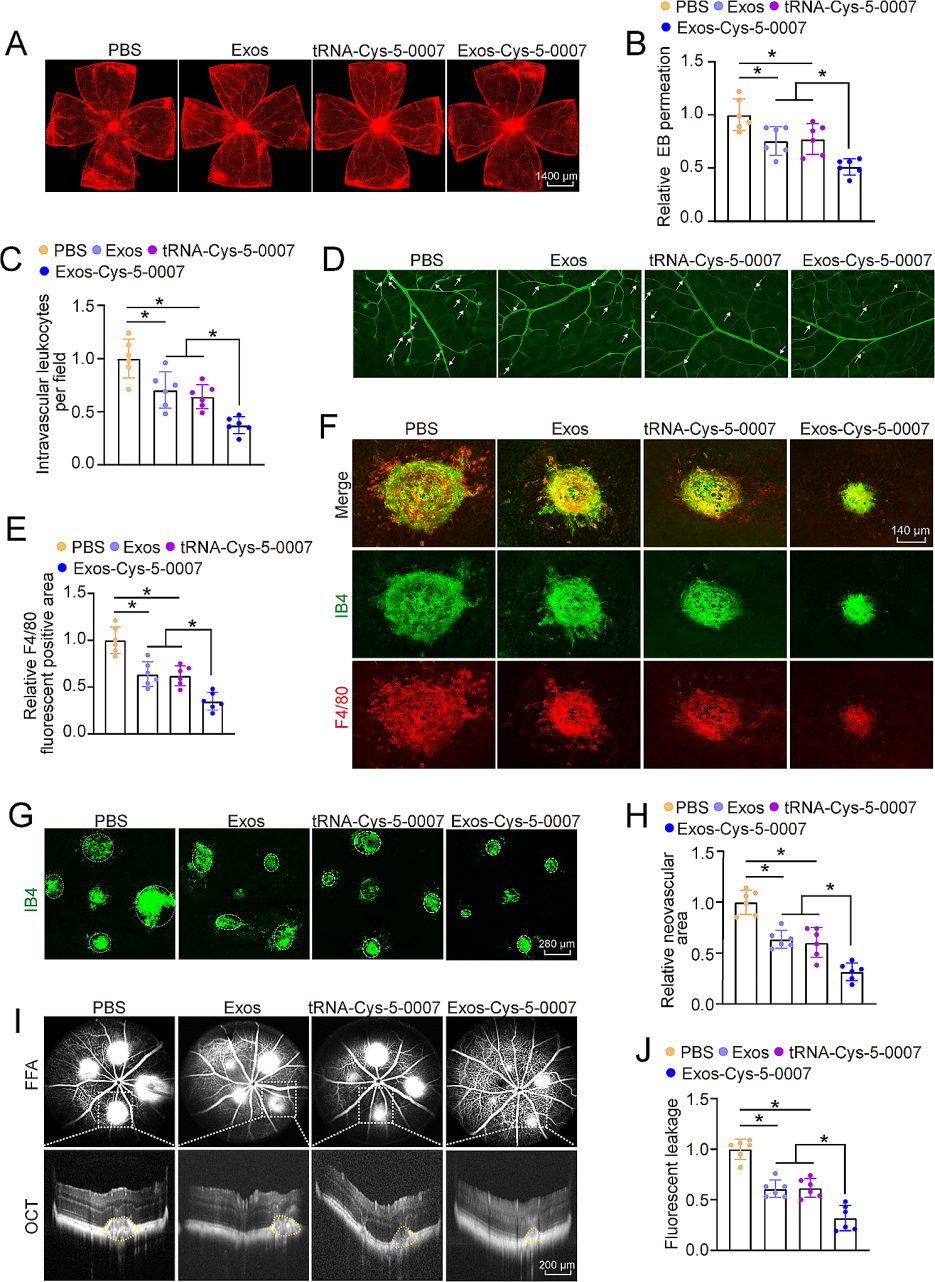
Exosomal formulation enhances the synergistic anti-angiogenic and anti-inflammatory efficacy of tRNA-Cys-5-0007. (**A**-**D**) Diabetic C57BL/6J mice (8-week-old, male) received intravitreous injections of PBS, exosomes, tRNA-Cys-5-0007, or Exos-Cys-5-0007. They were perfused with Evans blue dye for 2 h. The fluorescence signal of flat-mounted retinas was detected by a fluorescence microscope. The representative images were shown (**A** and **B**). The infiltrations of leukocytes in retinal vessels were detected by fluorescein-isothiocyanate (FITC)-coupled concanavalin A lectin perfusion assays. Adherent leukocytes (white arrows) were stained with FITC-conA (**C** and **D**). (**E**-**J**) CNV mice (8-week-old, male) received intravitreous injections of exosomes, tRNA-Cys-5-0007, Exos-Cys-5-0007, or PBS. On day 14 after laser injury, CNV lesions were stained with IB-4 (green) and F4/80 (red) to label CNV lesions and macrophages (E and F, *n* = 5). RPE/choroid complexes were collected and stained with IB-4 to label CNV lesions. The representative images were shown. Green staining indicated CNV lesions; dashed line indicated CNV areas (G and H). FFA and OCT images of CNV mice following treatment with exosomes, tRNA-Cys-5-0007, Exos-Cys-5-0007, or PBS and quantitative fluorescence leakage areas for spots were shown. The non-CNV group was taken as the control group. The part circled in yellow delineated the bulges in subretinal spaces (I and J). The data were presented as the means ± SD. *n* = 6 mice per group; *P < 0.05; One-way ANOVA followed by Tukey’s multiple comparisons test

In DR model, intravitreal injections of exosomes or tRNA-Cys-5-0007 were effective in mitigating diabetes-induced retinal vascular leakage. Notably, exosomal formulation of tRNA-Cys-5-0007 (Exos-Cys-5-0007) achieved the best treatment efficiency in mitigating retinal vascular leakage (Fig. 7A and B). Intravitreal injections of exosomes or tRNA-Cys-5-0007 could reduce the number of adherent leukocytes within retinal capillaries. Exosomal formulation of tRNA-Cys-5-0007 (Exos-Cys-5-0007) exhibited the best efficiency in reducing the number of adherent leukocytes compared with the other groups (Fig. 7C and D). In OIR model, intravitreal injections of exosomes or tRNA-Cys-5-0007 were effective in reducing both the neovascular areas and avascular areas. Particularly, exosomal formulation of tRNA-Cys-5-0007 (Exos-Cys-5-0007) demonstrated the highest efficiency in anti-angiogenic effects (Fig. S7A and S7B).

In CNV model, intravitreal injections of exosomes or tRNA-Cys-5-0007 had the anti-inflammatory effects characterized by reduced F4/80/IB4 co-expressed regions, while exosomal formulation of tRNA-Cys-5-0007 (Exos-Cys-5-0007) exhibited better anti-inflammatory effects (Fig. 7E and F). IB4 staining, fundus fluorescein angiography (FFA), and optical coherence tomography (OCT) were conducted to investigate the anti-angiogenic and anti-inflammatory efficacy of tRNA-Cys-5-0007. IB4 staining showed that intravitreal injections of exosomes or tRNA-Cys-5-0007 partially reduce CNV lesions compared to PBS group after 14-day treatment. Particularly, exosomal formulation of tRNA-Cys-5-0007 (Exos-Cys-5-0007) has proven more effective in reducing CNV lesions, suggesting its better treatment efficacy for CNV lesions (Fig. 7G and H). FFA and OCT images further demonstrated a marked reduction in fluorescence leakage following intravitreal injections of exosomes or tRNA-Cys-5-0007. Exosomal formulation of tRNA-Cys-5-0007 exhibited the highest inhibitory effects on CNV formation. OCT images of retinal cross-sections demonstrated that obvious ruptures and bulges in subretinal spaces were observed in CNV group and symptom mitigation was received following intravitreal injections of exosomes, tRNA-Cys-5-0007, or Exos-Cys-5-0007. Exosomal formulation of tRNA-Cys-5-0007 exhibited the optimal treatment efficiency in subretinal lesions.

## Discussion

Pathological angiogenesis is a common feature in ocular neovascular diseases such as PDR, AMD, and ROP, leading to vision loss and potential blindness [40]. Currently, clinical treatment options for retinal vascular diseases include laser surgery, intraocular injection of anti-VEGF drugs, vitrectomy, and other techniques [41]. Prolonged use of anti-VEGF medications presents challenges as a significant number of patients do not respond well to this treatment, develop resistance, or experience varying degrees of retinal atrophy [9]. Nucleic acid drugs are generally easier to administer compared to peptides or proteins, and they are well-suited for targeting both extracellular and intracellular targets [42, 43]. In this study, we have highlighted the capacity of a small RNA derived from transfer RNA, specifically tRNA-Cys-5-0007, to selectively target retinal vessels and mitigate pathological angiogenesis, vascular leakage, and inflammatory responses.

The eye relies on both the inner retinal circulation and choroidal circulation to receive oxygen, nutrients, and remove waste [44]. However, in diseased conditions characterized by elevated glucose levels, hypoxia, inflammation, and pro-angiogenic factors, pathological angiogenesis, encompassing both choroidal and retinal angiogenesis, can occur [45]. Endothelial cells (ECs) play a crucial role in maintaining vessel integrity and act as a barrier to maintain tissue homeostasis. When ECs undergo dysfunction, it significantly contributes to the progression of pathological angiogenesis [39, 46]. Our study elucidated the pivotal role of tRNA-Cys-5-0007 as a central regulator in ocular angiogenesis. Through a comprehensive analysis focused on ocular neovascular diseases, we observed a decline in tRNA-Cys-5-0007 levels during ocular angiogenesis across three distinct models and in endothelial cells under pathological conditions. Furthermore, our research underscored the significance of tRNA-Cys-5-0007 in angiogenesis in vivo, as its inhibition led to heightened abnormal ocular angiogenesis in both the retina and choroid. Conversely, overexpression of tRNA-Cys-5-0007 attenuated pathological angiogenesis and inflammatory reactions in retinal or choroidal tissues. Moreover, tRNA-Cys-5-0007 demonstrated potent inhibitory effects on the proliferation, migration, sprouting, and tube formation capabilities of retinal endothelial cells. These findings underscore the substantial impact of tRNA-Cys-5-0007 across various ocular neovascular diseases and highlight its potential as a promising therapeutic target.

tsRNAs play important roles in both the physiological conditions and pathological conditions [47, 48]. They frequently associate with Ago2 and engage in interactions with the 3′-UTRs of messenger RNAs through miRNA-like base pairing, resulting in the degradation of target mRNAs and/or the inhibition of their translation [49, 50]. We identified VEGFA and TGF-β1 as target genes through bioinformatics predictions, and their interaction with tRNA-Cys-5-0007 was validated using a dual-luciferase reporter assay. VEGFA is a major regulator of pathological angiogenesis and is also involved in ocular angiogenesis. It binds to tyrosine kinase (TK) receptors, VEGFR-1 (Flt-1), and VEGFR-2 (KDR/Flk-1). VEGFA promotes angiogenesis by stimulating endothelial cell proliferation, migration, vascular permeability, and angiogenic initiation [51]. Increased levels of VEGFA contribute to vascular leakage and the development of ocular angiogenesis. TGF-β1, one of the isoforms of TGF-β family, is widely studied and plays a significant role in ocular neovascular diseases [52]. Dysregulation of TGF-β signaling leads to increased production of extracellular matrix (ECM), vascular permeability, and destabilization [53, 54]. TGF-β1 may stimulate angiogenesis by promoting choroidal endothelial cell proliferation, inducing VEGF-A secretion from retinal pigment epithelium (RPE), or mediating inflammation through macrophages. Pathological TGF-β signaling contributes to the breakdown of blood-retinal barrier, vascular damage, neuroinflammation, and pathological angiogenesis [52]. Indeed, the significant downregulation of VEGFA and TGF-β1 expression levels by tRNA-Cys-5-0007 supplementation underscores their critical role as key targets in pathological neovascularization. Consequently, it’s understandable that overexpression of tRNA-Cys-5-0007 leads to a decrease in ocular vascular leakage and a reduction in ocular inflammation. These findings highlight the effectiveness of targeting VEGFA and TGF-β1 through tRNA-Cys-5-0007 as a promising therapeutic strategy for managing ocular neovascular diseases.

In the field of small RNA therapeutics, off-target silencing is a well-established concern. While tRNA-Cys-5-0007 effectively silences target genes like VEGFA and TGF-β with high specificity, it may inadvertently silence the unintended transcripts as well. This selectivity arises because off-target transcripts typically lack perfect complementarity with the guide strand, particularly within the “seed region” (nucleotides 1–8), akin to how microRNAs recognize their targets. However, the impact of off-targeting on the specificity of tRNA-Cys-5-0007 necessitates further investigation. To comprehensively address off-target effects, future research should explore strategies such as novel gene carriers or chemical modifications of small RNAs to enhance delivery and mitigate potential off-targeting [53, 54].

As natural carriers, exosomes have attracted great attentions as drug delivery vehicles due to their low immunogenicity and favorable safety profile. They have excellent stability with a long half-life in the bloodstream and can be easily taken up by cells [55, 56]. Additionally, their nanoscale size allows them to traverse blood-ocular barriers effectively [57]. Exosomes can be administered through vitreous cavity to reach the inner and outer retinal layers, or through peribulbar or retrobulbar regions across the ocular walls, providing a safer and more efficient drug delivery method compared to systemic intravenous injection [55]. Moreover, engineered exosomes can inhibit inflammatory responses and specifically target inflammatory sites [29]. In this study, we demonstrate that both exosomes and tRNA-Cys-5-0007 treatments effectively alleviate ocular vascular dysfunction and suppress retinal inflammation. The exosomal formulation of tRNA-Cys-5-0007 enhances the synergistic anti-angiogenic and anti-inflammatory effects of tRNA-Cys-5-0007. Importantly, exosome-mediated delivery of tRNA-Cys-5-0007 exhibits no significant cytotoxicity or tissue toxicity. These results highlight the potential of exosomes as drug delivery vehicles and the promising role of Exos-Cys-5-0007 in anti-angiogenic and anti-inflammatory treatment for experimental ocular angiogenesis.

Indeed, while our study provides valuable insights into the role of tRNA-Cys-5-0007 and exosomes in ocular neovascularization, there are still limitations that need to be addressed. Longer-term studies would be necessary to understand the sustained effects and long-term outcomes of tRNA-Cys-5-0007 and Exos-Cys-5-0007 treatment. Analyzing different time points post-injury or treatment could reveal dynamic changes. Additionally, cell-type-specific binding and targeted delivery should be carefully considered to avoid non-target gene regulation, which could trigger unwanted side effects like toxic reactions or immune responses. In future studies, efforts should be made to chemically modify exosomes for improved small RNA delivery, enhancing their therapeutic targeting effects. This could involve engineering exosomes to enhance their stability, cellular uptake, and targeting ability, thereby improving the overall efficacy of tRNA-Cys-5-0007 delivery.

## Conclusions

This study provides valuable insights into the role of tRNA-Cys-5-0007 in ocular angiogenesis and its potential therapeutic applications. Overexpression of tRNA-Cys-5-0007 exhibits anti-angiogenic effects in vitro and reduces ocular vessel leakage, pathological angiogenesis, and ocular inflammation in vivo. The anti-angiogenic and anti-inflammatory effects of tRNA-Cys-5-0007 are mediated through the targeting of VEGFA and TGF-β1. The exosomal formulation of tRNA-Cys-5-0007 demonstrates synergistic anti-angiogenic and anti-inflammatory efficacy. Overall, this study highlights the potential of tRNA-Cys-5-0007 as a promising therapeutic target for ocular angiogenesis-related disorders. The development of an effective exosome-based delivery system for tRNA-Cys-5-0007 is a significant advancement in translating this research into clinical applications.

### Electronic supplementary material

Below is the link to the electronic supplementary material.

Supplementary Material 1

## Data Availability

The data and materials supporting this study are available within the paper and its Additional fles information. Additional information can be obtained from the corresponding author upon reasonable request.
