# Supplementary material for: Dual anti-angiogenic and anti-inflammatory action of tRNA-Cys-5-0007 in ocular vascular disease
Source: J Transl Med. 2024 Jun 12;22:562. doi: 10.1186/s12967-024-05338-w (PMC11167814; doi:10.1186/s12967-024-05338-w)
Supplement: Supplementary file 1 — Supplementary Material 1 [file 12967_2024_5338_MOESM1_ESM.pdf]

Supplemental data

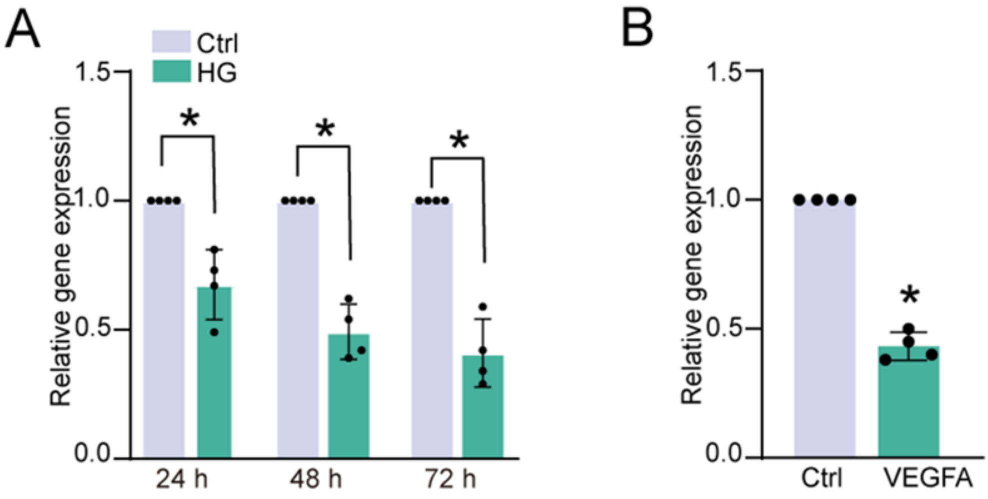

Supplemental Figure 1: tRNA-Cys-5-0007 expression is reduced following high glucose stress and angiogenic stress *in vitro*

(A) HRVECs were exposed to high glucose (30 mM) or left untreated (Ctrl) for 24 h, 48 h, and 72 h. The levels of tRNA-Cys-5-0007 expression were detected by qRT-PCR assays. (B) HRVECs were exposed to VEGFA or left untreated (Ctrl) for 24 h. The levels of tRNA-Cys-5-0007 expression were detected by qRT-PCR assays. The data were presented as means ± SD; n = 4; *$P < 0.05$.

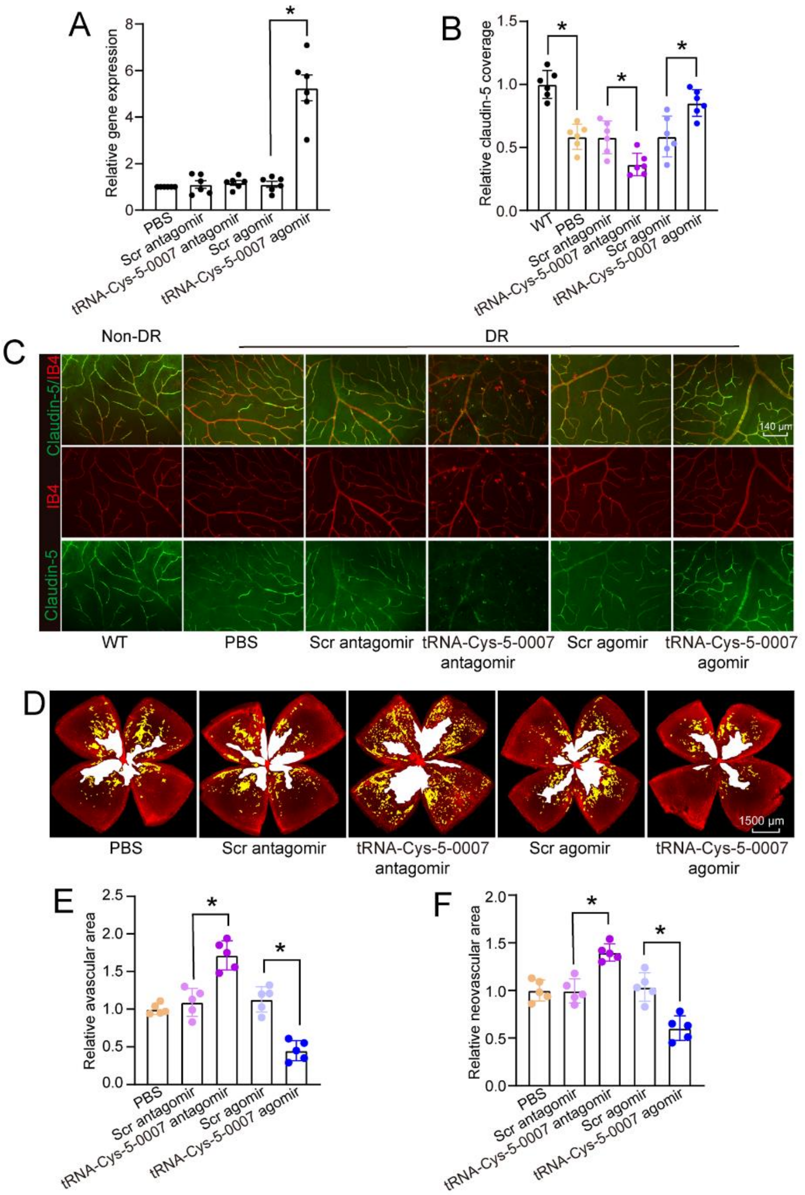

Supplemental Figure 2: tsRNA-Cys-5-0007 regulates retinal vascular dysfunction *in vivo*

(A) The mice received intravitreal injections of PBS, Scr agomir, tRNA-Cys-5-0007 agomir, Scr antagomir, or tRNA-Cys-5-0007 antagomir for 7 d. qRT-PCR assays were conducted to detected tsRNA-Cys-5-0007 expression. (B and C) Double-fluorescent staining of Claudin5/IB4 was conducted to detect the integrity of retinal vessel barrier. The representative images of IB4 (red) and Claudin-5-stained (green) retinal vessels were visualized by a fluorescence microscope (n = 6). (D-F) The neonatal C57BL/6J mice (7 d) were exposed to 75% oxygen for 5 days following intravitreous injections of PBS, Scr antagomir, tsRNA-Cys-5-0007 antagomir, Scr agomir, tsRNA-Cys-5-0007 agomir, and were then exposed to RA (21% oxygen) until P17. The retinas were collected on P17 and stained with Isolectin B4. Yellow staining indicated the neovascular area; white area indicated the avascular area (n = 5). The data were presented as means ± SD; *$P < 0.05$.

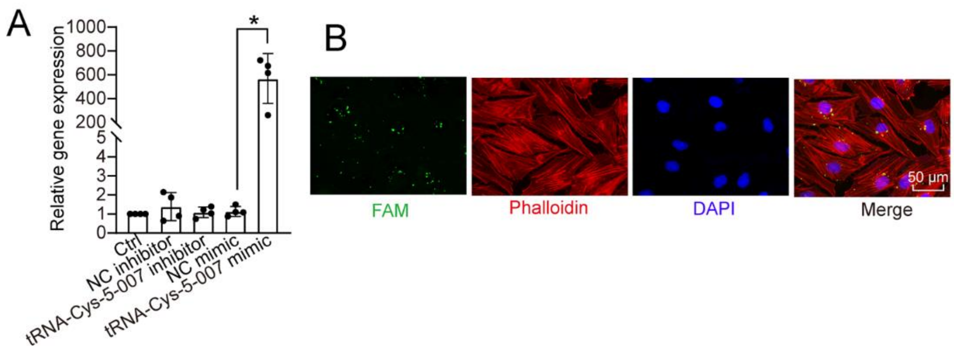

Supplemental Figure 3: Detection of transfection efficiency of tsRNA-Cys-5-0007 in HRVECs

(A) qRT-PCR assays were conducted to detect the levels of tRNA-Cys-5-0007 expression following transfection of negative control (NC) mimics, tRNA-Cys-5-0007 mimics, NC inhibitors, or tRNA-Cys-5-0007 inhibitors, or left untreated (Ctrl) for 24 h. qRT-PCR assays were conducted to detect tsRNA-Cys-5-0007 expression. (B) HRVECs were transfected with FAM labelled inhibitor (green) for 6 h. Cell nuclei were stained by DAPI (blue). Cell cytoskeletons were stained by Phalloidin (red). The representative images were taken by a fluorescence microscope. The data were presented as means ± SD; n = 4; *P < 0.05.

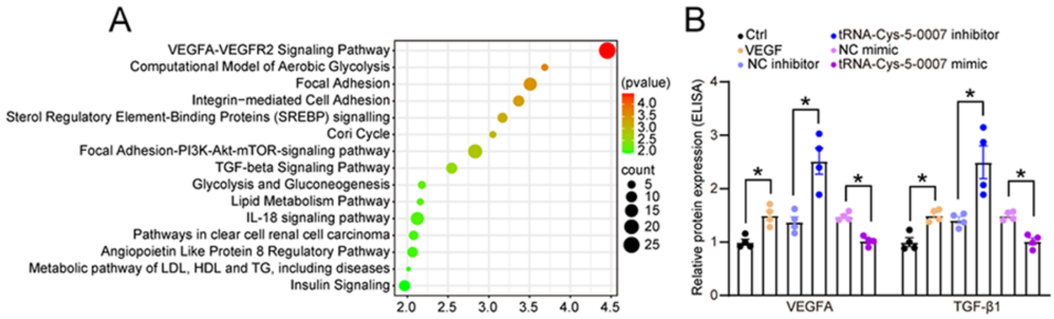

Supplemental Figure 4: tRNA-Cys-5-0007 regulates endothelial cell function by targeting VEGFA and TGF-β1

(A) The target genes of tsRNA-Cys-5-0007 were predicted by the tsRTar database. Pathways analysis of target genes was conducted by Wikipathways database. (B) The levels of VEGFA and TGF-β1 expression were determined by ELISA assays following transfection with negative control (NC) mimics, tsRNA-Cys-5-0007 mimics, NC inhibitors, tsRNA-Cys-5-0007 inhibitors, exposed with or without VEGF (Ctrl). The data were presented as means ± SD; n = 4; *P < 0.05.

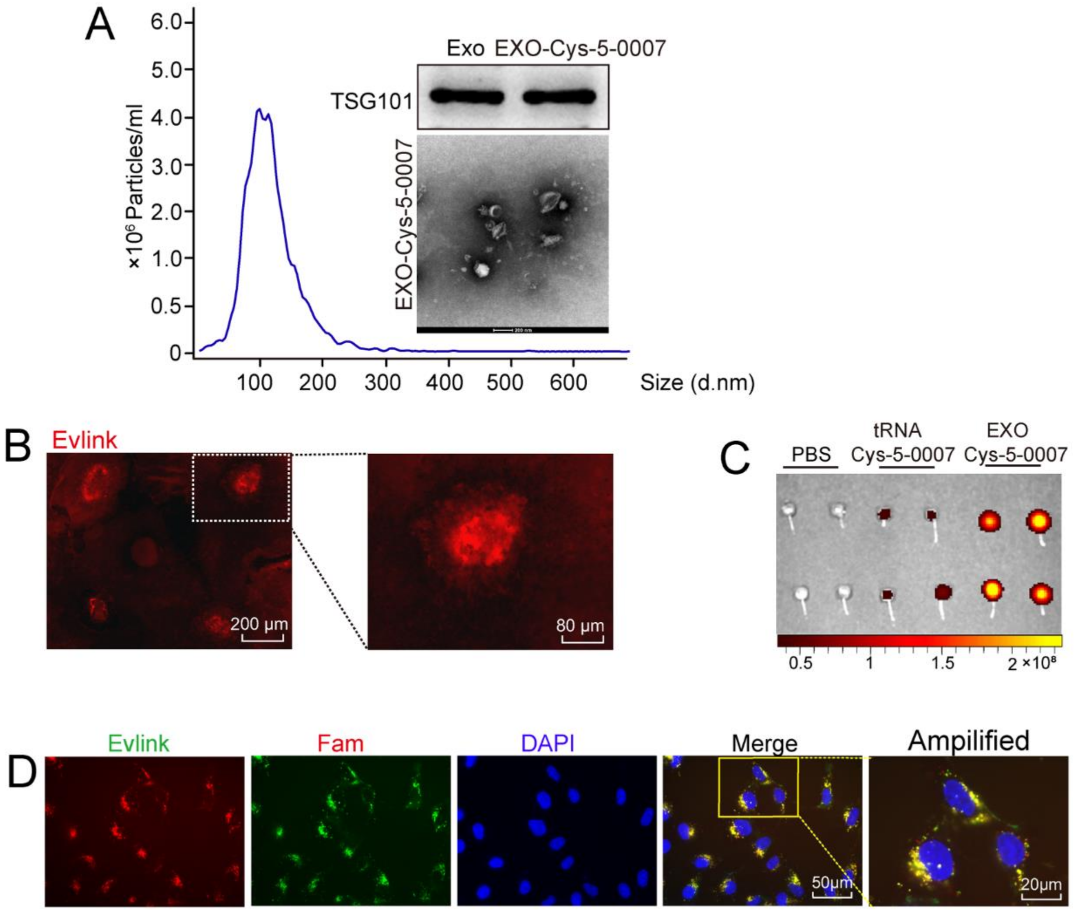

Supplemental Figure 5: Preparation of Exos-Cys-5-0007 and uptake of Exos-Cys-5-0007 *in vivo* and *in vitro*

(A) Exosomes were loaded with tRNA-Cys-5-0007 mimics by electroporation. The characterization of Exos-Cys-5-0007 were conducted, including the observation of morphology using a transmission electron microscope (TEM), detection of TSG101 markers using western blots, and determination of the diameters using nanoparticle tracking analysis. (B) Evlink labeled exosomes were injected immediately following laser photocoagulation. The uptake of hUC-MSCs cells-derived exosomes in CNV model were observed at day 3 following injection by a fluorescence microscope. (C) Mice bioluminescence imaging was conducted to visualized Cy3-labeled tRNA-Cys-5-0007 using the *in vivo* imaging system. Cys-labeled tsRNA-Cys-5-0007 or Exos-Cys-5-0007 was intravitreally injected. The ocular fluorescence was imaged at day 3 following injection. (D) After incubation with HRVECs for 24 h, the co-localization between Fam (green)-labeled tRNA-Cys-5-0007 mimics and Evlink (red) labeled exosomes was detected in HRVECs. Cell nuclei were stained with DAPI (blue).

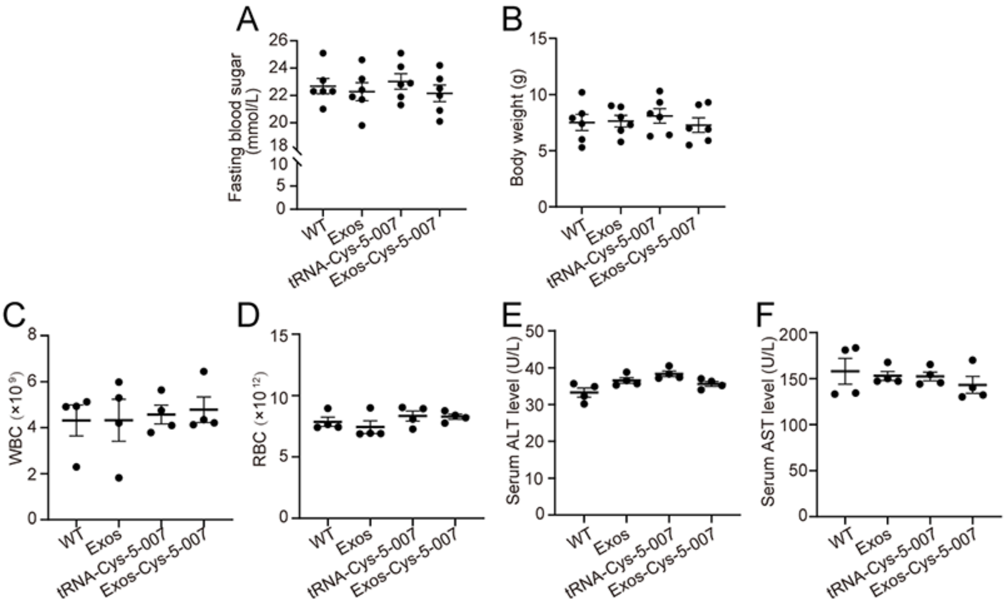

Supplemental Figure 6: Systemic safety evaluation of Exos, tRNA-Cys-5-0007, or Exos-Cys-5-0007 treatment

(A-F) The mice received intravitreal injections of PBS, Exos, tRNA-Cys-5-0007, or Exos-Cys-5-0007 for 7 d. The potential systemic toxicity was evaluated by blood sugar levels (A, n = 6), weight change (B, n = 6), blood cell count (C and D, n = 4), and serum liver enzymes (E and F, n = 4) at day 7 after injection. The data were presented as means ± SD; **P* < 0.05.

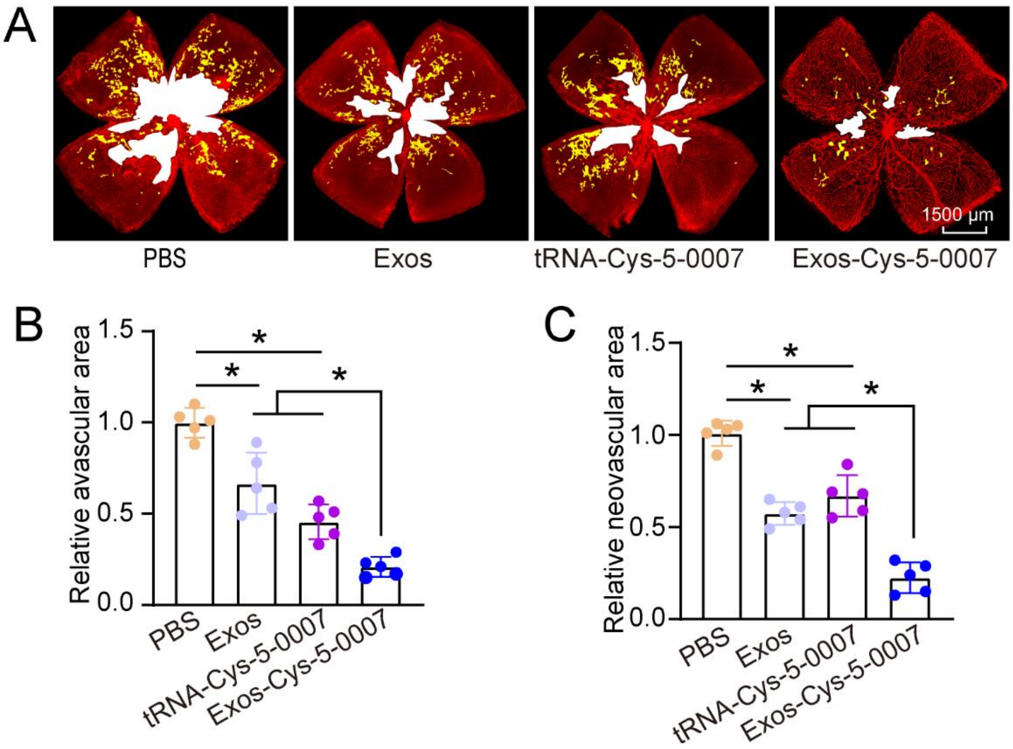

Supplemental Figure 7: Exosomal formulation enhances synergistic anti-angiogenic efficacy of tRNA-Cys-5-0007 in OIR model

(A-C) The neonatal C57BL/6J mice (7 d) were exposed to 75% oxygen for 5 days following intravitreous injections of PBS, exosomes, tRNA-Cys-5-0007, Exos-Cys-5-0007, and were then exposed to RA (21% oxygen) until P17. The retinas were collected on P17 and stained with Isolectin B4. Yellow staining indicated neovascular areas; white regions indicated avascular areas. The representative images were shown (n = 5). The data were presented as means ± SD; *$P < 0.05$.

Table S1. Primer sequences used for qPCR assays for mRNAs

Gene	Forward primer sequence (5'-3')	Reverse primer sequence (3'-5')
VEGFA	GGAGGAGGGCAGAATCATCAC	CTTGGTGAGGTTTGATCCGC
TGF-β1	TGGTGGAAACCCACAACGAA	GAGCAACACGGGTTCAGGTA

Table S2. Primer sequences used for qPCR assays for sncRNAs

Gene	Forward primer sequence (5'-3')
tRNA-Cys-5-0007	GGCGGGTATTGCTCAGGG
U6	CCTGCTTCGGCAGCACA

*Universal reverse primer provided by the reverse transcription kit.

Table S3: RNA oligonucleotide sequences

Gene	Sequence (5'-3')
tRNA-Cys-5-0007 mimic	GGGGGUAUAGCUCAGGGGUA
tRNA-Cys-5-0007 inhibitor	UACCCCUGAGCUAUACCCCC
NC mimic	AAGCGCUGGGAGUGUGGGAU
NC inhibitor	AAGCGCUGGGAGUGUGGGAU
tRNA-Cys-5-0007 agomir	GGGGGUAUAGCUCAGGGGUA
tRNA-Cys-5-0007 antagomir	UACCCCUGAGCUAUACCCCC
Scramble agomir	GCGAGGUUGAGCUGGAUAGG
Scramble antagomir	GCGAGGUUGAGCUGGAUAGG

*The agomir, antagomir, and their respective scrambled negative control RNAs were defaulted methylation and cholesterol modification. The mimics, inhibitors, agomir, and antagomir were provided by RiboBio (Guangzhou, China)

Supplemental materials and methods

Tube formation assay

The 24-well plates were coated with 35 μL of Matrigel (354230, Corning, USA) per well and incubated at 37°C for 1 h. The pre-treated HRVECs cultured on 6-well plates were harvested. After gelation, the matrigel was overlaid with 500 μL of medium containing 4×10^4 of HRVECs per well. These cells were cultured in a CO_2 incubator (37°C, 5% [v/v] CO_2, 95% humidity). A branched endothelial network was visualized by a light microscope (IX73P1F, Olympus, Japan). The quantification of capillary tube formation was conducted using Image J.

Transwell migration assay

After transfection, endothelial cells were harvest and adjusted to a concentration of 1×10^5 cells/mL in ECM. The 8 μm cell culture inserts (353097, BD Falcon, USA) were placed in a 24-well plate with tweezers. Then, 600 μL of ECM complete medium was added to the lower chamber, while 200 μL of cell suspension was added to the upper chamber. After 12 h, the cells in the lower chamber were fixed by methanol for 15 min. The transwell chamber was then placed upside down for 10 min for air dry. Next, the cells were stained 0.2% crystal violet (C805211, Macklin, China) for 5 min. The crystal violets were washed away with ddH_2O. These non-migrated cells in the upper chamber were gently removed using a cotton swab. The images were obtained under a light microscope.

5-ethynyl-2'-deoxyuridine (EdU) assay

BeyoClick™ EdU Cell Proliferation Kit with Alexa Fluor 488 (C0071S, Beyotime,

China) was used for the detection of cell proliferation following the manufacturer's instruction. Briefly, the cells were plated into 24-well plate and allowed to grow until 80% cell density. EdU solutions were added to incubate for 2 h. Next, the cells were fixed with 4% PFA and permeabilized with a buffer (0.3 % Triton X-100) for 15 min. Subsequently, the reaction mix was added to label EdU and incubate for 30 min. The nuclei were labeled with DAPI. The images were obtained by a fluorescence microscope and EdU-positive cells were counted.

Endothelial cell spheroid-based sprouting angiogenesis assay

Endothelial cells were harvested and adjusted to the concentration of 2×10^4 cells/mL. Cell suspension was mixed with 1.2% methocel stock solution (419273, Sigma, USA) and dripped onto the lid of a petri dish. The dish was incubated at 37 °C for 24 h. Subsequently, the hanging drops containing spheroids were gently washed off using PBS. The spheroids were then centrifuged at 200 *g* for 3 min to form the pellets to discard the supernatants. 2 mL mixture of methocel stock solution containing 20% FBS and collagen I (354236, Corning, USA) was added and mixed to avoid bubbles. Next, 1 mL of spheroid mixture was added to a 24-well plate and incubated at 37℃ for 30 min to promote solidification. Finally, the spheroids were cultivated at 37℃ for 24 h and imaged.

Evans blue assay

Evans blue assays were used to detect the breakdown of blood-retinal barrier. The first step was to prepare Evans blue solution (30 g/L, K29136, Kehbio, China) using sterile saline and add 400 U/mL of heparin sodium salt (1170GR001, BioFroxx,

German) for anti-coagulation. The solution was shaken overnight at room temperature, filtered with a 0.22 μm sterile filter and stored at 4℃ for the preservation. To perform Evans blue assays, the animals were anesthetized and secured in an upward position with the abdomen facing up. The hair from the inner thighs was removed and a longitudinal incision was made in the inner thigh skin. Blunt dissection of the fascia was then performed to expose femoral vein. Then, the animals were slowly injected with Evans blue solution (30 mg/kg) using an insulin syringe. After 30-min circulation, the eyeballs were gently enucleated and fixed with 4% PFA for 30 min. The anterior segment, lens, and vitreous were removed away from the eyecups and the retinas were dissected free from choroid-sclera complexes and cut into four-leaf clover shape under a stereo surgical microscope. EB extravasation from retinal vessels was observed under a fluorescence microscope. Retinal permeability was evaluated by detecting the concentration of EB dye in the plasma from iliac artery. After measuring wet weight, the retinas were thoroughly dried in a desiccator. To extract EB dye, the retina was incubated with 120 μL of formamide at 70 °C for 18 h. The extract was centrifuged at 12, 000 *g* for 30 min at 4 °C. Absorbance at 620 nm (blue signal) and 740 nm (background subtracted) of the supernatant was detected by the spectrophotometric method. Concentrations of EB dye was calculated based on the corresponding standard curve in formamide. The concentration of EB dye in blood sample was measured in the same manner. Retinal vascular permeability was calculated using the equation as shown below.

$$\frac{\text{Retinal Evans blue concentration } (\mu g) / \text{Retinal wet weight } (g)}{\text{Blood Evans blue concentration } (\mu g) / \text{Plasma } (\mu l) \times \text{Circulation time } (h)}$$

Retinal leukocytosis assay

The adhesion of leukocytes to retinal vessels was detected as previously described. Briefly, the mice were anesthetized and positioned with their abdomen. The chest cavity was opened using the scissors to expose the heart. A 20 G perfusion tube was inserted into the aorta and 10 mL of PBS was perfused to remove these nonadherent blood cells. Subsequently, 10 mL of fluorescent isothiocyanate (FITC)-labeled concanavalin A (Con A) lectin (40 μg/mL, FL-1001, Vector Laboratories, USA) was perfused to label the adherent leukocytes and vascular endothelial cells. After that, the mouse was cannulated with 10 mL of PBS to remove the remaining unbound Con A. Then, the eyeballs were enucleated and fixed with 4% PFA for 30 min. After fixation, the anterior segment, lens, and vitreous were removed from the eyecups. The retinas were dissected free from the choroid-sclera complexes. Then, the retinas were cut into four-leaf clover shape under a stereo surgical microscope. Retinal flat mounts were imaged under a fluorescent stereoscope and total number of leukocytes adhered to the vessel walls per retina was counted.

Immunofluorescent staining of choroidal flat mounts

After anesthesia, the mice were euthanized by cervical dislocation. The eyeballs were removed and fixed in 4% PFA for 30 min. Then, the fascia and connective tissues on the surfaces of eyeballs were carefully peeled off. The anterior segment, lens, and vitreous were removed away from the eyecups. RPE choroid-sclera complex was

dissected and cut into four-leaf clover shape under a stereo surgical microscope. RPE-choroid-sclera complex was fixed in 4% PFA for 30 min and washed with PBS for three times. To facilitate permeabilization, the complex was permeabilized with PBS containing 5% BSA and 1% TritonX-100 at 37°C for 45 min, incubated with the F4/80 primary antibody (1:200, ab6640, Abcam, England) overnight at 4°C, and then incubated with the secondary antibody or Isolectin B4 (1:25; L2895, Sigma-Aldrich, USA) at room temperature for 2 h. RPE choroid-sclera complexes were observed under a fluorescent scope.

Immunofluorescent staining

After the mice were anesthetized and euthanized, their eyeballs were removed and fixed with 4% PFA for 30 min. Following fixation, the fascia, connective tissue, anterior segment, lens, and vitreous were carefully removed from the eyecups. Subsequently, the retinas were dissected free and cut into a four-leaf clover shape under a stereo surgical microscope. The dissected retinas were fixed again with 4% PFA and permeabilized using a solution containing 5% bovine serum albumin (BSA) and 1% Triton X-100 for 45 min at 37°C. For immunostaining, the retinas were incubated overnight at 4°C with claudin-5 antibody (1:200, sc-374221, Santa Cruz, USA). This was followed by the incubation with the secondary antibody and GS-IB4 Alexa Fluor™ 594 (1:50, I21413, Thermo Fisher, USA) at room temperature for 2 h. Finally, the retinas were imaged under a fluorescent microscope to visualize the distribution of claudin-5 and GS-IB4.

Subcellular fractionation assay

The Cytoplasmic & Nuclear RNA Purification Kit (NGB-21000, Norgen Biotek) was employed to extract both the nucleus and cytoplasmic RNAs. This kit allows for the separation of RNA fractions based on cellular localization. After RNA extraction, qPCR assays were conducted to detect the levels of tRNA-Cys-5-0007 in both the nucleus and cytoplasmic fractions. To ensure accurate normalization and comparison of RNA levels between the two fractions, β-actin and U6 were detected as endogenous controls for cytoplasmic and nucleus RNAs, respectively. qPCR analysis provides insights into subcellular distribution of tRNA-Cys-5-0007 and allows for the assessment of its potential role in different cellular compartments.

RNA fluorescence *in situ* hybridization (FISH)

The Ribo™ Fluorescence *in situ* Hybridization Kit (C10910, RiboBio, China) was used following the manufacturer's instruction. Cy3-labeled tRNA-Cys-5-0007 probe (RiboBio, China) sequences were designed as 5'-ITACCCCTGAGCTATACICICICIC-3' Cy3, IT= (locked nucleic acid) LNAT, IC=LNAC. The cells were observed and imaged under a fluorescent stereoscope.

Quantitative reverse transcription PCR (qRT-PCR)

Total RNAs were extracted from the cells using the FastPure Cell/Tissue Total RNA Isolation Kit (Vazyme, RC112-01, China). For the analysis of tRNA-Cys-5-0007, reverse transcription was performed using the microRNA Reverse Transcription Kit (EZBioscience, EZB-miRT4, USA) following the manufacturer's instruction. Subsequently, qPCR was conducted using the EZ-Probe qPCR Master Mix (EZBioscience, EZB-miprobe-R2, USA). Expression levels of all target genes were

normalized to the expression of U6. For mRNA detection, total RNAs were reverse-transcribed into complementary DNAs (cDNAs) using the PrimeScript RT Master Mix (RR037A, Takara Bio, Japan). The resulting cDNAs were then subjected to qPCRs using the SYBR Premix Ex Taq II (RR820A, Takara Bio, Japan) according to the manufacturer's protocol.

Western blot

The cells were lysed in RIPA lysis buffer (P0013B, Beyotime, China) supplemented with Complete ™ Protease Inhibitor Cocktail (04693116001, Roche, Switzerland). Protein concentration in the lysates was determined and normalized using the BCA method (23225, Thermo Fisher, USA). Protein samples were prepared with a five-fold loading buffer and separated by SDS-PAGE, after which they were transferred to PVDF membranes (IPVH00010, Merck Millipore, Germany). The membranes were then blocked and incubated overnight at 4°C with primary antibodies. The primary antibodies used were VEGFA (Dilution, 1:1000) and TGF-β1 (Dilution, 1:1000). Following primary antibody incubation, the membranes were incubated with horseradish peroxidase-conjugated secondary antibodies (A0201/A0208, Beyotime, China). Finally, the protein bands were visualized using a BeyoECL Plus kit (P0018S, Beyotime, China).

Enzyme-linked immunosorbent assay (ELISA) assay

After incubation at 37 °C for 24 h, the medium was collected and examined for cytokine and chemokine production with TGF-β1 ELISA Kit (E-EL-0162c, eBioscience, USA) and VEGF-A ELISA Kit (E-EL-H0111, eBioscience, USA)

according to the manufacturer's instruction.

RNA immunoprecipitation (RIP) assay

The cells were lysed using RIP lysis buffer, and the resulting lysates were incubated with magnetic beads conjugated with either anti-Ago2 (67934-1-Ig, Proteintech, China) or anti-IgG (30000-0-AP, Proteintech, China) overnight at 4°C. This incubation allowed for the immunoprecipitation of RNA-protein complexes, specifically those associated with Ago2 or IgG. Following the incubation, RNA was eluted and purified from the immunoprecipitated complexes. The enriched RNA, including tRNA-Cys-5-0007, was then subjected to qRT-PCRs. This approach facilitated the identification and quantification of RNA molecules that interact with Ago2, providing insights into the regulatory roles of tRNA-Cys-5-0007 within the RNA-induced silencing complex.

RNA pull down assay

The cellular proteins were extracted using IP lysis buffer and then incubated for 30 min. The lysates underwent centrifugation at 3, 000 *g* for 20 min at 4°C to obtain the total protein. The total protein was combined with a biotinylated probe specific to tRNA-Cys-5-0007 or a negative control probe (RiboBio, China). The mixture was left to incubate overnight at 4°C. Following overnight incubation, the protein-probe complexes were exposed to Streptavidin MagPoly Beads (SM017005, Smart-Lifesciences Biotechnology, China) at 26°C for 2 h. After washing to remove the non-specific bound proteins, the RNA-binding protein complexes were eluted. To prepare the eluted complexes for western blot, 5 × SDS-PAGE loading buffer (P0015,

Beyotime, China) was added, and the mixture was boiled for 25 min. The presence of the target protein in the pull-down complex was assessed. A portion of cell lysate was retained as the input control. For immunoprecipitation, the primary antibody employed was Ago2 antibody (1:1000, 67934-1-Ig, Proteintech, China). This approach allowed for the identification and characterization of protein interactions with tRNA-Cys-5-0007.

Luciferase reporter gene assay

The luciferase reporter assay was performed using the Dual Luciferase Reporter Gene Assay Kit (RG027, Beyotime, China). VEGFA 3'UTR-Wt and Mut, as well as TGF-β1 3'UTR-Wt and Mut sequences, were incorporated into the GP-miRGLO vector (GenePharma, China). These luciferase reporter vectors were then transfected with either tRNA-Cys-5-0007 mimics or negative control mimics. After a 48-h post-transfection period, the luciferase activity was quantified relative to *Renilla luciferase* activity using the Dual Luciferase Reporter Gene Assay Kit.

Detection of ocular drug distribution

To assess delivery efficiency, bioluminescence imaging was conducted on mice using the IVIS Spectrum In Vivo Imaging System (IVIS® Spectrum, PerkinElmer, USA). Cy5-labeled tRNA-Cys-5-0007 or Exos-Cys-5-0007 were intravitreally injected into ICR mice, which are an albino mouse strain suitable for imaging with IVIS. After 24 h, the mice were euthanized and their eyeballs were collected for imaging. To detect the distribution of exosomes in a laser-induced CNV model, Evlink-labeled exosomes were intravitreally injected following laser injury. Choroids were isolated three days

after injection for analysis.

Exosome uptake *in vitro*

HRVECs were seeded into 24-well plates at the density of 4 × 10^4 cells/well and cultured for 24 h. To detect the uptake of Exos by HRVECs, the isolated Exos (200 μg protein/150 μL DPBS) were labeled with CD63 (1:50, sc-5275, Santa Cruz, USA). The labeled Exos were then incubated at room temperature for 30 min and subsequently centrifuged at 100,000 *g* to remove excess antibody. HRVECs were incubated with 50 μg/mL labeled Exos in FBS-free medium for 24 h. Then, the medium was discarded and the cells were washed with DPBS (14190144, Thermo Fisher, USA) for 3 times. Next, the cells were fixed for 30 min at room temperature using 4% PFA (BL539A, Biosharp Biotechnology, China). The permeabilization of HRVECs was performed for 30 min at 37°C in DPBS containing 5% BSA (4240GR100, BioFroxx, German) and 0.1% TritonX-100. Subsequently, the cells were incubated overnight at 4°C with the secondary antibodies. After incubation, the cells were washed with DPBS for 3 times and the nuclei were labeled with DAPI (1:1500; C1002, Beyotime, China). The images were captured by a fluorescence microscope (IX73P1F, Olympus, Japan).

OCT and FFA assay

OCT and FFA assays were conducted using a Saris Multi-Modal Ophthalmic ImagingSystem for Animal (Robotrak, China). To dilate the pupils, 0.5% tropicamide with 5% phenylephrine was administered. To visualize blood vessels, the mice were intraperitoneally injected with 10% fluorescein sodium at a volume/weight ratio of 50 μL/20 g. Fluorescence accumulation images were captured during the late stationary

phase. FFA and OCT images were captured with a 30° angle of view. To quantify vascular permeability, Image J software was used for analysis.

TUNEL assay

TUNEL assays were conducted to detect retinal apoptosis. Following cardiac perfusion, the eyeballs were harvested for making frozen sections. These sections were then allowed to re-warm at 25°C for 30 min and washed with PBS for three times. Retinal apoptosis was detected using the TUNEL Apoptosis Detection Kit (Beyotime, C1098, China) according to the manufacturer's instruction. DNase included positive controls were performed using the Apoptosis Inducers Kit (C0005, Beyotime, China). The nuclei were labeled with DAPI and the images were captured by a fluorescence microscope.

Hematoxylin-eosin (H&E) staining

H&E staining was used to detect the change of retinal structure. The eyeballs were removed and preserved in paraffin, followed by cutting them into 5 μm sections. These sections underwent sequential alcohol dehydration and were cleared with xylene. Subsequently, they were stained with hematoxylin for 1 min and eosin for 30 sec. The changes of retinal structures observed under a light microscope.

Flow cytometry and Calcein-AM/PI double staining

Flow cytometry and Calcein-AM/PI double staining was conducted to detect cell apoptosis. After treating HRVECs with Exos for 24 h, Calcein-AM (22003, AAT Bioquest, USA) and PI (17517, AAT Bioquest, USA) were added to label the live and apoptotic cells at 37°C for 15 min in dark. The nuclei were labeled with Hoechst 33258

(Beyotime, C1011, China). The images were captured using a fluorescence microscope. For flow cytometry analysis, HRVECs were trypsinized and washed with PBS for 3 times. Annexin V Binding Buffer, Annexin V-FITC, and PI were added according to the manufacturer's instruction and incubated in dark for 15 min. The percentage of cell apoptosis was determined by flow cytometry.

ERG examination

The animals were dark adapted overnight and prepared for ERG recording under dim red light. To dilate the pupils, 0.5% tropicamide with 5% phenylephrine was administered. The mice were anesthetized and one drop of oxybuprocaine (0.4%) was applied for corneal anesthesia. The placement of the recording electrode was placed on the central cornea, while the reference needle electrode was placed behind the ears and the ground electrode in the tail. To prevent corneal desiccation, one drop of 1% carboxymethylcellulose was applied to the cornea. Following recording of a scotopic intensity series, the photopic flash responses were measured using increased light stimuli. The b-wave amplitude was measured from the trough of a-wave to the peak of b-wave. ERG recordings were performed using the Roland Consult Color Ganzfeld Q450C recording machine.

Hematology tests

White blood cell (WBC), red blood cell (RBC), serum aspartate transaminase (AST), and alanine transaminase (ALT) were analyzed by Wuhan Pinuofei Biological Technology Company.
